# Phage-plasmid-like elements are found throughout diverse environments and encode niche-specific functional traits

**DOI:** 10.1371/journal.pone.0350027

**Published:** 2026-05-29

**Authors:** James I. Mullet, Liqing Zhang, Amy Pruden, Connor L. Brown

**Affiliations:** 1 Department of Civil and Environmental Engineering, Virginia Tech, Blacksburg, Virginia‌‌, United States of America; 2 Department of Civil and Environmental Engineering‌‌, Massachusetts Institute of Technology, Cambridge, Massachusetts, United States of America; 3 Department of Computer Science, Virginia Tech, Blacksburg, Virginia‌‌, United States of America; 4 Department of Chemistry and Biosciences, Aalborg University, Aalborg, Denmark; Tianjin University, CHINA

## Abstract

Phage-plasmids are unique mobile genetic elements that function as plasmids and temperate phages. While it has been observed that such elements often encode antibiotic resistance genes and defense system genes, little else is known about other functional traits they encode. Further, no study to date has documented their environmental distribution and prevalence. Here, we performed genome sequence mining of public databases of phages and plasmids utilizing a random forest classifier to identify phage-plasmids. We recovered 5,712 unique phage-plasmid-like genomes from a remarkable array of disparate environments, including human, animal, plant, fungi, soil, sediment, freshwater, wastewater, and saltwater environments. The resulting genomes were used in a comparative sequence analysis, revealing functional traits/accessory genes associated with specific environments. Host-associated elements contained the most defense systems (including CRISPR and anti-CRISPR systems) as well as antibiotic resistance genes, while other environments, such as freshwater and saltwater systems, tended to encode components of various biosynthetic pathways. Interestingly, we identified genes encoding for certain functional traits, including anti-CRISPR systems and specific antibiotic resistance genes, that were enriched in phage-plasmid-like elements relative to both plasmids and phages. Our results highlight that phage-plasmid-like elements are found across a wide-array of environments and likely play a role in shaping microbial ecology in a multitude of niches.

## Introduction

Vehicles of horizontal gene transfer (HGT), such as plasmids and phages, are key drivers of prokaryotic adaptation and evolution [1,2]. In this regard, their role in the mobility of accessory genes, i.e., genes that are not required for the basic life cycle of a mobile genetic element (MGE), is of particular interest [2,3]. MGEs can carry accessory genes encoding diverse traits that may be advantageous to their hosts, including antibiotic resistance genes (ARGs), virulence factors, defense systems such as CRISPR-Cas, metal resistance genes (MRGs), and toxin-antitoxin systems, among many others [3]. Such genes can provide hosts with resiliency in the face of changing selective pressures. While MGEs are typically discussed as independent classes [2,4], there is an emerging awareness of their composite nature, and even inter-element interactions (e.g., conflicts occurring between MGEs within individual bacterial hosts) [5–8]. For example, some phages, plasmids, and integrative and conjugative elements carry genes encoding defense systems that interfere with the function of co-infecting MGEs [1]. Prokaryotic defense systems like these are hypothesized to be acquired through selective bacteriophage predation and have been demonstrated to cluster with and potentially increase the spread of ARGs [9,10]. The carriage of defense systems by MGEs can result in complex ecological and evolutionary dynamics within their host and can significantly alter the community dynamics of microbial populations [1,9].

Phage-plasmids (P-Ps) are a newly characterized class of MGE that occupy a unique place in the landscape of prokaryotic genomic elements. These elements can be generally described as temperate (i.e., integrated) phages that retain the ability to replicate in a plasmid-like manner as extra-chromosomal DNA as part of their host life cycle [11]. A small set of P-Ps have been shown experimentally to employ a unique combinatorial replication strategy, leveraging both phage lysis and reinfection and the multi-copy number potential of plasmids [11,12]. Additionally, P-Ps have been shown to transfer ARGs, certain defense systems, and additional accessory genes from both phages and plasmids [12–14]. With supporting research indicating that P-Ps are significant promoters of genetic exchange between phages and plasmids, the composition and diversity of their accessory genomes remains a key knowledge gap [14]. On the other hand, challenges remain in characterizing the ecology of P-Ps purely from sequencing data. For example, some P-P-like elements have lost essential phage genes and have undergone conversion to single element life cycles. However, this does not preclude the possibility that genes encoded by inactive prophages retain some cryptic function. In sum, the unique biology of P-Ps and these phage-plasmid-like elements (PPLEs) makes the question of their accessory genome particularly intriguing, with the potential for distinct infection, spread, and HGT strategies. P-Ps thus represent a new and poorly understood dimension of microbial community dynamics and a distinct transfer pathway for accessory genes such as AMR or CRISPR-Cas systems.

However, to date, the environmental distribution of P-Ps and PPLEs have not been determined. Indeed, whether P-Ps are common features of microbial communities or merely rare oddities that emerge in specific niches has not yet been ascertained. This limited examination into P-P biological diversity becomes critical to understand as phages and plasmids independently possess unique functional variation across different environments [15,16]. Understanding the diversity of P-Ps across these environments can provide improved insights into the impacts and potential interactions these elements have in the exchange of accessory genes between microbial species.

## Results

### PPLEs are prolific in public databases of phages and plasmids‌‌

**Fig 1:**
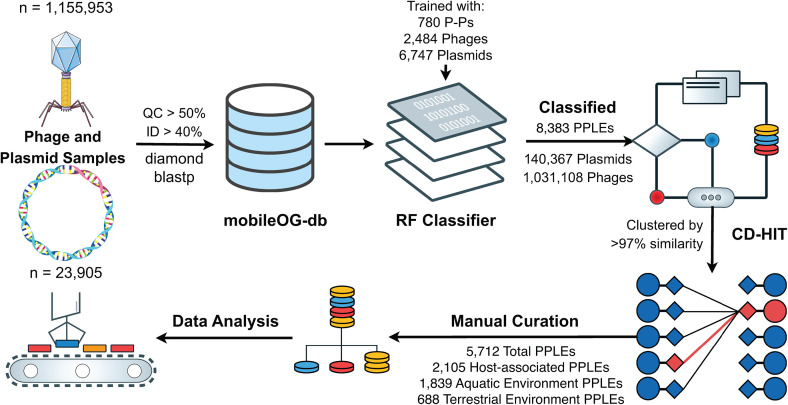
Filtering and identification of phage-plasmid-like elements from publicly-available phage and plasmid genomes. The genomes from the three phage databases (n = 1,155,953) and one plasmid database (n = 23,905) were processed against mobileOG-db to identify MGE-related hallmark genes [21]. The genomes were then reclassified into phages (n = 1,031,108), plasmid (n = 140,367), and phage-plasmid-like element (n = 8,383) using a random forest classifier that identifies PPLEs using phage and plasmid hallmark proteins. The phage plasmids were then clustered (n = 5,712) to remove identical genomes and manually curated by the associated source location of the classified genomes.

We analyzed 1,179,858 genomes from databases of plasmids and phages (PLSDB [17], GPD [18], MGV [19], and IMG/VR [20]) for PPLEs using a random forest classifier. The features of the model included the number of hallmark protein hits to each class of MGE (bacteriophage, plasmid, integrative elements, insertion sequences, and multiple), the associated mobileOG-db major categories for each protein, and the number of total proteins and open reading frames for each genome (S1 Table; Fig 1 in S1 File; Text 1 in S1 File) and were trained on (10,289 genomes from [Pfiefer et al.]) [11,21]. The classifier had a recall/precision/F1 score for the PPLE class specifically of 80.1%, 70.0%, and 74.7% based on the training dataset of 780 P-Ps. To further validate the model, we compared classification results from a dataset of 1,416 P-Ps, achieving a recall of 85.8% (Text 1 in S1 File) [21]. This classifier was designed to limit false positives from metagenomic sequences, and the results potentially underestimate the exact number of P-Ps in this dataset.

This model was employed to generate a conservative, high-confidence set of PPLEs, which was especially relevant because of our usage of IMG/VR v4.0, a database of phage genomes derived primarily from environmental metagenomes [20]. Importantly, it should be emphasized that these are P-P-like elements as they are inferred from metagenomic sequences and have not been experimentally confirmed to possess dual life style attributes.

**Fig 2:**
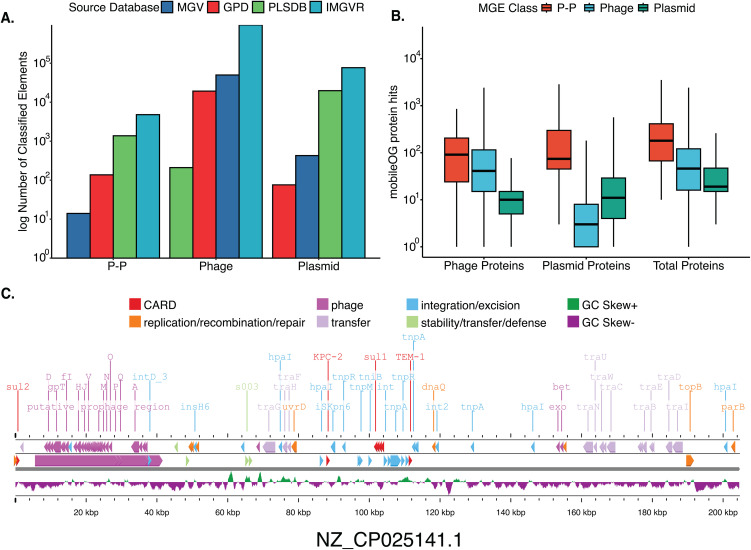
Phage-plasmid-like elements (PPLEs) are prolific in databases of plasmids and phages. **(A)** Number of classified MGEs of each element class from the four respective databases before dereplication. **(B)** The hybrid nature of PPLEs are reflected in the patterns of mobileOGs. **(C)** Illustration of a phage-plasmid-like element from PLSDB (id = NZ_CP025141.1) depicted using Proksee including Phigaro, Prokka, mobileOG-db, CARD, and GC Skew annotations [17,21,23–27]. All unlabeled or unclassified proteins were removed from this figure.

The final P-P-like dataset examined in this study was composed of 5,712 dereplicated genomes with 137 from GPD, 13 from MGV, 4,395 from IMG/VR, and 1,167 from PLSDB (Fig 2A) [17–20]. This includes 1,318 phage-plasmids previously identified from prior works (Text 3 in S1 File) [11,21]. PLSDB was predicted to contain several phage genomes (0.8% of PLSDB sequences) and phage databases, such as IMG/VR, were found to harbor many plasmid sequences (Fig 2A) [17,20]. This is not necessarily surprising, as accuracy of plasmid and phage identification can be affected by both low-quality annotated databases and the inherent bias of tools and datasets that specifically classify only one type of MGE. Prior studies have shown that plasmid classification tools can be prone to misidentifying phages as plasmids and, likewise, phage identification tools sometimes misidentify plasmids as phages [21,22]. These inherent biases of analyses targeting a single class of MGE highlight the value of predicting multiple MGE classes simultaneously. Additionally, our model supported a P-P-like status for 42 of 45 P1-like Plasmids reported by Pfiefer et al. (PMC10879196), contradicting previous findings. Examining these more closely, we found that 90.5% (n = 38) had an intact prophage region with 7.1% (n = 3) having a questionable or incomplete prophage region and only 1.5% (n = 1) having no detectable prophage region. However, we also found that several of these P1-like elements were also classified as P-Ps by tyPPing, highlighting the difficulty of differentiating these closely related elements.

Metadata across the PPLE set was harmonized to group PPLEs according to the environment from which the original sample was sourced: terrestrial (n = 688); aquatic (n = 1,839); host-associated (n = 2,105); and unclassified (n = 1,080) (Table in S1 Table) (Fig 1). Comparative analysis of mobileOGs (i.e., MGE hallmark genes) highlighted distinct profiles of gene content across phages, plasmids, and PPLEs (Fig 2B). These profiles were consistent with expectations in that PPLEs encoded more phage genes than plasmids (Fisher exact test; median 88 genes vs. 8 genes; p < 0.001); more plasmid genes than phages (Fisher exact test; 55 genes vs. 0 genes; p < 0.001); and more total genes than both phages and plasmids (Fisher exact test; 179 PPLE genes vs. 46 phage genes vs 19 plasmid genes; p < 0.001) (Fig 2B). In addition, PPLEs were found to have larger average genome sizes than either phages or plasmids, as has been reported previously in studies that examined a smaller dataset of PPLEs (Figs 4 and 12 in S1 File). We further examined the differences in genome size across different environments and found that human and animal sourced PPLEs possessing the largest mean genome sizes (Fig 5 in S1 File).

### PPLEs are associated with disparate hosts and ecological niches

Examining the putative hosts of PPLEs can provide insight into the ecology of PPLEs across distinct environmental niches. A compilation of source database metadata was used in tandem with sequence analysis to identify predicted host taxonomy, plasmid incompatibility groups, phage morphology, and the source environment of the PPLE genomes. A putative viral taxonomic classification was obtained for 5,148 genomes classified into viral taxonomic families using geNomad [22]. The bacterial host taxonomy was obtained with 58.8% of PPLEs (n = 3,371) receiving a phylum-level classification (Table in S1 Table).

**Fig 3:**
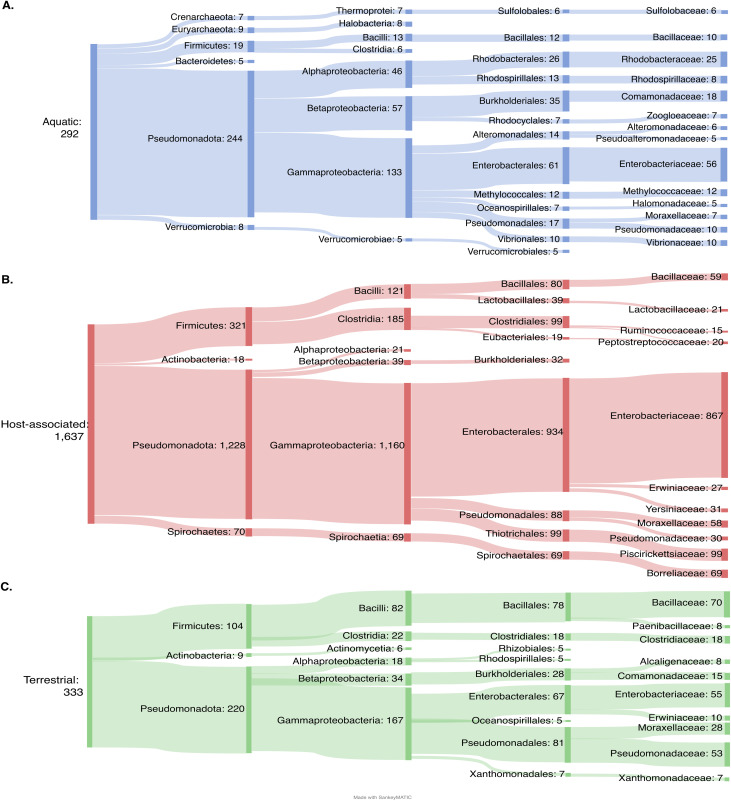
Classification and distribution of PPLEs according to the reported source organism and environment. Examination of the relative abundance of PPLE host predicted taxa for aquatic **(A)**, host-associated **(B)**, and terrestrial **(C)** genomes. The predicted host phyla, class, order, and family of each respective source location are included in each subfigure [28]. The predicted host taxa for any PPLEs without reported environmental source locations were excluded from this analysis. Any infrequent taxa that were <1% abundance in the respective environmental location were not included in this figure.

We next investigated the prokaryotic hosts of PPLEs across different environments. The most commonly predicted bacterial host phyla across all environments were *Pseudomonadota*, *Bacteroida*, and *Clostridia*. The aquatic PPLEs possessed the highest diversity in predicted prokaryotic host phyla, including several phyla (*Verrucomicrobia*, *Crenarchaeota*, and *Euryarchaeota*) exclusively associated with aquatic PPLEs (Fig 3). Further examination revealed differences in the class-level taxonomy of the PPLE bacteria. Within the *Pseudomonadota* phylum, *Gammaproteobacteria* was the most common predicted bacteria class, particularly in host-associated PPLEs (95.1% host-associated PPLEs, 76.3% terrestrial PPLEs, and 56.5% of aquatic PPLEs). *Alphaproteobacteria* and *Betaproteobacteria* classes were associated with more terrestrial and aquatic PPLEs (4.8% host-associated PPLEs, 23.6% terrestrial PPLEs, and 46.2% of aquatic PPLEs). The terrestrial PPLEs in *Gammaproteobacteria* were primarily from the *Pseudomonadales* order, while aquatic PPLEs were affiliated with a broader array of bacterial carriers (Fig 3). Host-associated PPLEs were predominately carried by *Enterobacteriaceae* (71.2% of *Pseudomonadota* -associated hosts), a family that includes many enteric Gram negatives of clinical relevance, such as *Escherichia*, *Salmonella*, and *Shigella* (Fig 3) [29]. The *Enterobacteriaceae* bearing PPLEs were more frequently found among the host-associated PPLEs compared to both the aquatic (22.9%) and terrestrial (25.0%) *Pseudomonadota* bearing PPLEs (Fig 3).

We next examined the taxonomy of the PPLEs themselves. An analysis of the updated ICTV family classifications and plasmid systems was performed using geNomad and MobMess, respectively [22,30,31]. *Caudoviricetes* represented the dominant viral order across all environments, with 1.6% of aquatic PPLEs assigned classifications from *Megaviricetes* – an order containing giant viruses [32] (Fig 6 in S1 File). At the family level, few PPLEs could be classified using geNomad, but it was noted that the most frequently detected viral family was *Kyanoviridae* (n = 70), which was only found among aquatic PPLEs [22].

To interrogate the potential host ranges of the PPLEs, we analyzed the conserved plasmid regions of the various PPLEs and their association with specific prokaryotic hosts. Approximately 37.8% of the PPLEs were characterized into known plasmid systems. The results were supportive of narrow phylum-level host ranges among the PPLEs (Fig 8 in S1 File). However, host range was more variable at the genus level. We next examined whether specific proteins might be associated with putative host-ranges of the PPLEs, and whether it was likely the phage or the plasmid driving the observed host range. We annotated the cluster containing the PS5|PS235|PS682 plasmid backbone using mobileOG-db (Fig 9 in S1 File). This cluster contains PPLEs associated with the *Enterobacter* and *Salmonella* host genus. We found overall these PPLEs have a syntenically conserved plasmid replication module (repA, dnaE, dnaG, parB, recA) as well as shared phage infection-associated genes (smc, roi, thyA, V) (Fig 9 in S1 File). There was variability in the PPLEs’ integration/excision genes with *Enterobacter* associated PPLEs containing additional insertion sequences and transposase (IS3, tnpR, ISSen4) (Fig 9 in S1 File).

### PPLEs encode diverse and niche-specific accessory functions

The broad distribution of PPLEs across disparate environments led us to question what traits PPLEs might encode across a correspondingly wide variety of ecological niches. We next investigated the accessory genome of PPLEs, including ARGs, metabolism-related genes, metal resistance genes, defense systems, toxin-antitoxin systems, anti-CRISPR systems, and virulence factors.

**Fig 4:**
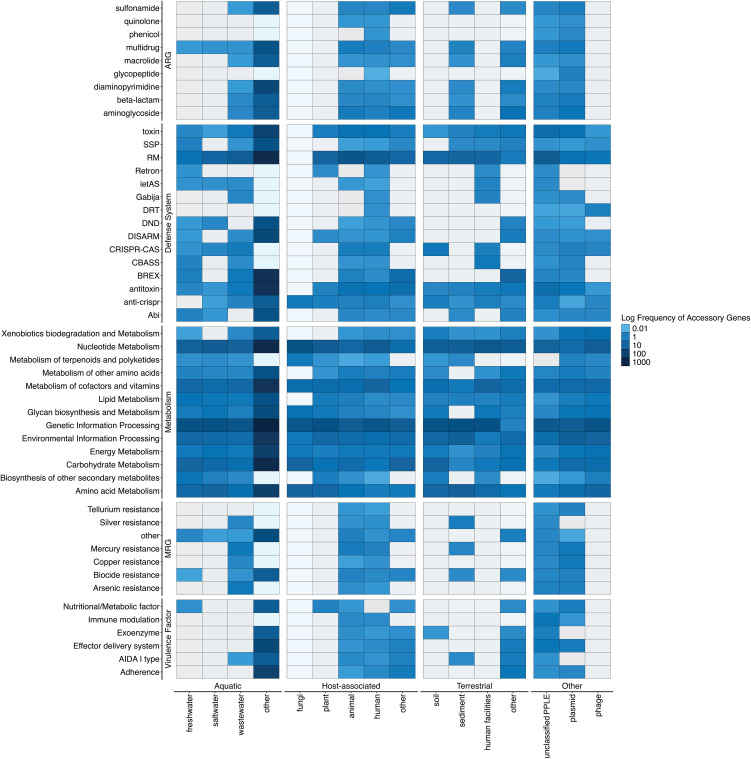
Comparative analysis of key accessory genes found to be carried by the PPLEs across a diverse range of source environments. The accessory genes were grouped into virulence factors, metal resistance, metabolism, defense systems, and antibiotic-resistance genes (ARGs). These genes were grouped into associated functional categories as shown in the supplementary tables. It was noted that both the toxin-antitoxin genes identified from TADB and the anti-CRISPR genes classified from Anti-CRISPRdb v2.2 were grouped with the defense system genes for visual purposes [33,34]. Only accessory gene categories with at least 25 hits were included in the figure above. The values were taken from the log10 of the relative frequency of the genes compared to the total number of accessory genes found in each element source location. The plasmid and phage categories comprise 500 random phages and plasmids, capturing differences between the various class of MGEs and acting as experimental baseline controls for comparing phages, plasmids, and PPLEs.

Accessory gene content of PPLEs was relatively unchanged within each of the distinct environments from which the PPLEs originated (Fig 4). When comparing PPLEs to phage and plasmid accessory genes, PPLE accessory gene profiles were most similar to those of plasmids (Kruskal-Wallis and post hoc Dunn test; p = 5.30 x 10^−1^), with very few accessory genes found among phages relative to plasmids and PPLEs (Kruskal-Wallis and post hoc Dunn test; p = 1.15x 10^−9^) (Fig 4). However, it was noted that the PPLEs had enriched anti-CRISPR genes compared to phages and plasmids (Fisher exact test; 240 PPLE genes vs. 5 phage genes vs 1 plasmid genes; p < 0.001). While most ARGs, MRGs, and virulence factors likely predominately originated from plasmid sources, it is also possible that phages still contribute to certain metabolism and defense system accessory genes among PPLEs.

### Diversity within the unique accessory genomes of phage-plasmid-like elements

**Fig 5:**
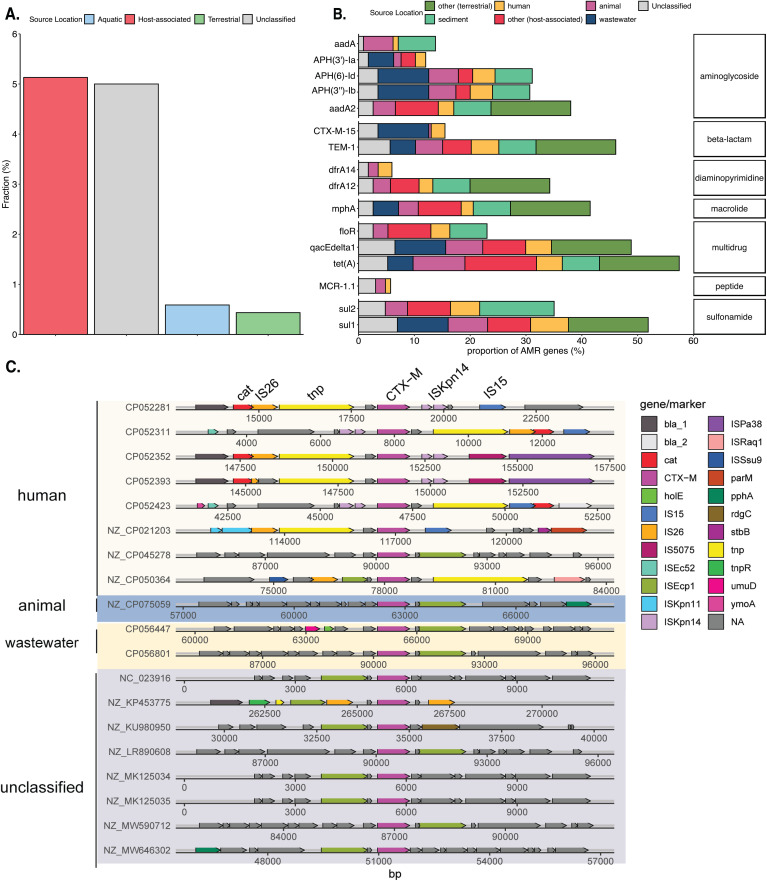
Diversity and distribution of ARGs among PPLEs of various origin. **(A)** Frequency of all ARGs found in PPLEs originating from each source environment. **(B)** Frequency of common antibiotic resistance genes (ARGs) carried by the PPLEs relative to the total identified ARGs in each source environment. Only source environments possessing >8 unique ARGs were included in the figure. **(C)** Gene-to-gene alignment of the CTX-M-15 ARG grouped by the respective source environment of the phage-plasmid-like elements [35].

We sought to further characterize the diversity among the unique PPLE accessory genomes and to assess additional differentiating features and trends among their profiles. First, we analyzed the differences between PPLE and plasmid ARG gene distributions. Similar to prior research, it was noted that PPLEs possess ARGs less frequently than plasmids. However, some ARGs, including cpxA, EcoI_emrE, and CTX-M-142, were enriched in PPLEs compared to plasmids (Fisher exact test; p < 0.01) (Fig 10 in S1 File). We found that several of the most common ARGs are associated with Class I integrons, including sul1, aadA2, and qacEdelta1 (Fig 5a). While approximately 5% of the host-associated PPLEs contained ARGs, the aquatic and terrestrial environment phage-plasmid-like elements appeared to be more depleted in the number of ARGs [36]. It was noted that the PPLEs associated with wastewater environments contained a few ARGs possessing the CTX-M-15 gene (Fig 5b). The CTX-M family of extended-spectrum beta-lactamases are among the most common causes of clinically-observed third-generation cephalosporin resistance [37]. Through the visualization of genetic contexts surrounding CTX-M-15, we found a conserved region that was encountered in PPLEs encountered across several examined source environments (Fig 5c).

**Fig 6:**
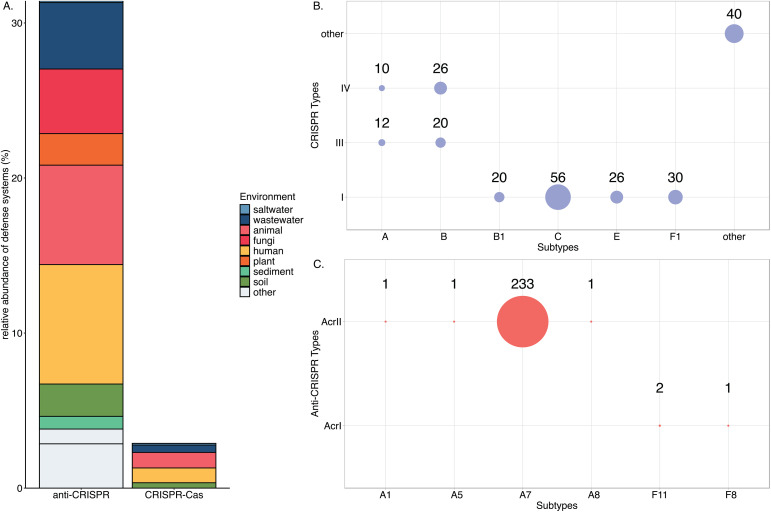
Analysis of the diversity and frequency of CRISPR-Cas and anti-CRISPR systems. **(A)** Relative abundance of anti-CRISPR and CRISPR-Cas systems encountered among unique PPLE genomes reconstructed from each of the respective source environments (Relative Abundance = PPLE genomes containing defense system from a respective source location/ Total PPLE genomes from respective source environments). **(B)** Distribution and occurrence of total CRISPR-Cas gene subtypes in PPLEs. “Other” includes systems that could not be classified into one single category or which were classified as a category other than the five primary classes of CRISPR-Cas systems. **(C)** Prevalence and abundance of anti-CRISPR system genes in PPLEs. Only subtypes found in the PPLEs are displayed in the figure.

Because of the hybrid-like status of P-Ps and potentially PPLEs as having both phage and plasmid type genes, an intriguing question is whether they utilize distinct defensive and offensive systems for interelement competition. We assessed the diversity of defense system genes including both CRISPR and anti-CRISPR systems. From this examination, PPLEs were found to possess more anti-CRISPR systems compared to CRISPR-Cas systems (Fig 6a) across all environments. CRISPR-Cas defense systems were frequently found in host-associated and terrestrial PPLEs, with lower abundance among the aquatic PPLEs (Fig 6). Most environments were characterized by even abundance of both classes of defense systems. However, some samples only recorded examples of one defense system class, such as animal host-associated PPLEs that possessed only CRISPR-Cas systems and fungi and plant PPLE genomes that carried anti-CRISPR systems.

We determined that only one PPLE (NZ_CP063966.1) possessed both a CRISPR-Cas system and anti-CRISPR system (Fig 11 in S1 File). This indicates that PPLEs typically utilize only one of these defensive or offensive strategies for limiting additional MGE co-infection [38,39]. The reduced variation of both systems in PPLEs was also noted. CRISPR-Cas systems genes were only found in Class I (n = 132), Class III (n = 32), and Class IV (n = 36) among the five major categories. Interestingly, the predominant anti-CRISPR system genes detected was the AcrIIA7 (n = 233), one of the most abundant anti-defenses CRISPR-associated inhibitors [40]. These results demonstrate that PPLEs can carry CRISPR-Cas and anti-CRISPR systems in various environmental sources, however, these defense systems appear to be most commonly encountered in host-associated PPLEs.

After examining the unique contributions of ARGs, defense‌‌ systems, virulence factors, and MRGs to phage-plasmid-like element accessory genes, it appeared that many of these genes are relatively consistent in their distribution across all environments from which the PPLEs were derived (Figs 17 and 18 in S1 File). The metabolic accessory genes were then examined to further investigate how this trend could impact other accessory gene functions (Fig 13 in S1 File). It was noted that the host-associated PPLEs possessed higher abundances of ARGs, certain defense systems, virulence factors, MRGs, and specific metabolic pathways such as pyrimidine metabolism, drug resistance, and cofactor and vitamin biosynthesis (Fisher Exact Test with a Benjamini-Hochberg correction; p < 0.001) (Fig 13 in S1 File). The freshwater and saltwater PPLEs contained enriched macrolide biosynthesis, photosynthetic genes and unique nucleotide metabolic pathways such as polyketide sugar biosynthetic pathways (Fisher Exact Test with a Benjamini-Hochberg correction; p < 0.001) (Fig 13 in S1 File).

## Discussion

Here, we investigated the functional repertoire of accessory genes and the ecological diversity of PPLEs. PPLEs were found to inhabit a wide range of environments and exhibited notable genetic variation, with evidence suggesting that most accessory genes are derived from plasmids [11,13,14]. PPLE encoded accessory genes included a diverse arsenal of ARGs, CRISPR-Cas systems, virulence factors, and metabolism genes. While prior research primarily demonstrates that P-Ps and PPLEs possess most accessory genes at rates intermediate to both phages and plasmids, we found evidence that some accessory gene elements are disproportionately associated with PPLEs [11,13,14]. Specifically, we found that anti-CRISPR systems and some ARGs [cpxA, EcoI_emrE, and CTX-M-142] were enriched in these elements (Fig 10 in S1 File, Fig 6). With our developing understanding of MGE competition (e.g., plasmids containing CRISPR-Cas systems that may target bacteriophages), it raises questions about the role of P-Ps in such interactions [ 1,41]. Prior work has shown that some phages bearing anti-CRISPR systems have density-dependent protection from CRISPR-Cas, suggesting a role for cooperation and/or co-infection in the defense mechanism [42]. Furthermore, it has been observed that P-Ps can exploit the replication machinery of plasmids to achieve a plasmids’ relatively high copy number potential [12]. This replication strategy could allow for higher phage densities, thus potentiating anti-CRISPR systems.

PPLE accessory gene content differed across environments. We examined various functional genes to investigate whether PPLEs confer traits that assist their prokaryotic hosts in adapting to their local environments. While these are not an exhaustive list of potential accessory genes, they are among the most important in understanding the ecology of PPLEs and their relevance to human health. We found that the distributions of these accessory genes varied significantly across environments. Host-associated PPLEs were enriched with defense systems, ARGs, virulence factors, and MRGs compared to aquatic PPLEs with increased abundances of intermediate secondary metabolic pathway genes. Many of these accessory genes appeared to be conserved, but the frequency varied depending on the environments from which these elements were recovered (e.g., Figs 5, 6). The overall trends of accessory genes appear similar to prior studies investigating plasmid gene diversity, although future works should investigate the differences between plasmid and phage-plasmid-like element accessory genes [36,43]. The variability in accessory gene content among PPLEs suggests that these elements might occupy unique niches within microbial communities depending on their environments.

PPLE genomic variation has the potential to alter microbial communities. Through the diversity of accessory gene content in host-associated, aquatic, and terrestrial-sourced PPLEs, we found a wide array of biologically-relevant accessory genes. These elements are prone to recombination and genetic exchange with other MGEs, making them of particular interest when considering their accessory genomes [14]. These unique biological features with the diverse array of accessory genes highlight the importance of further study into these elements [11–14,43]. Our results suggest that PPLEs offer notable genetic diversity and complexity that may impact MGE and bacterial evolution. The inherent variability of their hosts, viral genes, plasmid components, and functional genes these elements possess can play a significant role in shaping the recombination and HGT events in microbial populations. Understanding and potentially monitoring P-P and PPLE populations offers potential benefits to mechanistic understanding of the recombination and transmission of accessory genes such as ARGs, MRGs, and virulence factors, contributing to their overall spread. The P-P and PPLE accessory genomes should be studied further to fully understand how these elements spread this diverse assortment of accessory genes.

## Methods

### Data acquisition and processing

The complete genomes of 33,595 plasmids were retrieved from PLSDB, 19,510 genomes from GPD, 52,958 genomes from MGV, and 1,416,547 genome and associated fragments from IMG/VR databases [17–20]. An additional 8,248 plasmids, 2,256 phages, and 780 P-Ps were obtained from Pfeifer et al. for training the random forest classifier [11]. We removed genomes smaller than 10 kb to remove potentially fragmented genomes and genomes larger than 300 kb to avoid megaplasmids and chromatids. The information regarding the appropriate virus taxonomy, sampling source location, and additional information was collected from the metadata from PLSDB, GPD, MGV, and IMG/VR sources [17–20]. All analyses were conducted in Python (https://www.python.org/) unless otherwise stated.

### Annotation of Protein sequences

The genomes from PLSDB, GPD, MGV, IMG/VR, and Pfeifer et al. were processed with Prodigal (v2.6.3) using the (-p) meta setting to generate open reading frames [11,17–20,43]. The open reading frames were aligned to predicted protein sequences using diamond blastp (v4.6.8) using a minimum identity of 40%, minimum query coverage of 50%, maximum e-score of 1 x 10^−5^, and k value of 15 [43]. The less stringent settings allowed for the acquisition of more diverse phage species to ensure a high identification of all MGEs using mobileOG-db (Beatrix v1.6) [21]. This database provides an inclusive and diverse distribution of MGE protein sequences, which allows for a robust analysis of MGEs.

### Identification of phage-plasmid-like elements (PPLEs)

A random forest classifier was trained using the outputted results from the protein alignments using mobileOG-db [21]. The features utilized in the classifier included the number of protein hits to bacteriophages, integrative elements, insertion sequences, plasmids, and multiple MGE class proteins. In addition, the associated mobileOG-db major categories of the proteins (phage, integration/excision, replication/recombination/repair, transfer, and stability/transfer/defense) were included with the total number of proteins and ORFs found in each genome [21]. The Pfeifer et al. paper used several classification techniques including identifying P-Ps from literature sources, plasmid HMMs found in phages, and plasmids with identified phage-specific profiles for classifying P-Ps due to the limited known P-Ps prior to their work [11]. This paper utilizes the prior data obtained to train this classifier with the now known quantity of P-Ps. The model’s training began by performing ten randomized training sets using approximately 20% of samples as test data and 80% as training data. The random forest classifier had a max decision depth of 8 and used entropy as the criteria measurement. The performance results from the ten randomized trials were averaged to examine the effectiveness of the random forest classifier. The classifier achieved an average accuracy of 95.4% and a false positive rate of 2.9%. The testing data consisted of approximately 160 P-Ps, 500 phages, and 1350 plasmids, while the training data contained 620 P-Ps, 2,000 phages, and 5,400 plasmids [11]. The PLSDB, MGV, GPD, and IMG/VR genomes were then classified using the trained random forest classifier to identify whether each element was a plasmid, phage-plasmid, or bacteriophage [17–20]. To remove any potential giant viruses that remained in the dataset, we aligned the PPLEs against the Giant Virus Database and removed sequences with >80% percent identity [44]. CD-HIT-EST v4.6.8 were utilized to cluster the sequences and remove sequences with < 97% sequence similarity [45]. To compare between different clustering tools, MMSeqs2 v15.6f452 was used to cluster sequences at 95% percent identity and 85% query coverage [46]. The PPLEs were then examined using CompareM to compare the average nucleotide identity between the samples to compare the sequence similarity after clustering [47]. PHASTEST v3.0 was used to compare the prophage regions of potential P1-like Plasmids and P-Ps [48].

### Manual curation of source location

The classified phage-plasmid-like genomes were cross-referenced with the source database metadata to determine additional information regarding the source locations, host-range predictions, and taxonomy for additional analysis. The PPLEs were then categorized by environmental source location into the following categories: aquatic, terrestrial, host-associated, and unclassified environments. These categories were separated into more unique categories according to the exact location of the genomes, including the subcategories of saltwater, freshwater, wastewater, other aquatic genomes, soil, sediment, human facilities, other terrestrial, human, fungi, animal, plant, other host-associated genomes, and unclassified genomes. Genomes designated as others had designated source locations but were too generalized to classify the genomes further correctly. Phage-plasmid-like elements with undocumented source locations were cross-referenced with NCBI BioSample to classify the elements further, but genomes that still could not be classified were designated as other. All genomes with no metadata source locations or metadata with ambiguous locations were removed from source location analysis.

### Data analysis

The taxonomy of the PPLEs was classified using the associated source metadata from the respective databases. Due to the limited phage taxonomy, the viral taxonomic classifications were classified using geNomad (v1.5.2) [22]. The plasmid systems for PPLEs were identified using MobMess (v0.0.0), and P-P classifications were obtained using tyPPing [30,49]. The study further identified the key accessory genes of the phage-plasmid-like elements, including ARGs, defense systems, toxin-antitoxin systems, metabolism genes, metal resistance genes, and virulence factors. The defense systems were identified using PADLOC (v.1.1.0) classification tool [50]. The phage-plasmid-like genomes were processed through GhostKoala to extract the KEGGs from the Reconstruction Mapper function for identifying the metabolic genes [51,52]. Microbe Annotator (light-v2.0.5) was used to identify complete or partially complete KEGG Module pathways from the specific PPLEs using the blast settings [51,53]. These pathways were classified if the PPLEs contain 50% of the required genes for a specific biosynthesis pathway.

The virulence factors, metal resistance genes, anti-CRISPR genes, and the toxin-antitoxin systems were classified by processing the phage-plasmid-like elements using Diamond blastp (v4.6.8) against the VFDB genes from set A, the BacMet2 Predicted dataset, Anti-CRISPRdb (v2.2) database, and the TADB (v.2.0) database with query coverage of 80%, percent identity of 90%, and e-score of 1x10^-5^ [33,34,44,54,55]. The classified phage, plasmid, and PPLE genomes were queried against CARD (v3.0.7) with a minimum identity of 80% and an e-value<10^−10^ [26]. The phage-plasmid-like genomes were then processed through EggNOG-Mapper (v2) to get the associated PFAMs, and COGs for the additional PPLE analysis [56–58]. A random selection of 500 phages and 500 plasmids were isolated from the prior classified phage and plasmids classifications. These genomes were processed utilizing the same tools as the phage-plasmid-like elements to determine the accessory genes found in these genomes. The graphical analysis was performed using R (https://www.r-project.org/), draw.io (http://draw.io/), and bioicons (https://bioicons.com/).

## Importance‌‌

Phage-plasmids are a class of mobile genetic element which retain aspects of both phages and plasmids. However, whether phage-plasmids represent merely a rarity or are instead distinct players in horizontal gene transfer and other important ecological processes has remained a mystery. Here, we document that these elements are encountered across a broad range of distinct environments and encode niche-specific functional traits, including the carriage of antibiotic biosynthesis genes and both CRISPR and anti-CRISPR defense systems. These findings highlight phage-plasmid-like elements as an important class of mobile genetic element with diverse roles in multiple distinct ecological niches.

## Supporting information

S1 Table Benchmarking, training, and PPLE associated metadata.
(XLSX)

S2 Table Description of PPLE annotations from CARD, KEGG, VFDB, and BacMet.
(XLSX)

S3 Table Description of PPLE annotations from TADB, PADLOC, anti-CRISPR and P1-Plasmid analysis.
(XLSX)

S1 File Supplementary methods.
Contains additional data, methods, benchmarking information and results.
(DOCX)
