# Supplementary material for: Phage-plasmid-like elements are found throughout diverse environments and encode niche-specific functional traits
Source: PLoS One. 2026 May 29;21(5):e0350027. doi: 10.1371/journal.pone.0350027 (PMC13221032; doi:10.1371/journal.pone.0350027)
Supplement: S1 File — Supplementary methods. Contains additional data, methods, benchmarking information and results. (DOCX) [file pone.0350027.s004.docx]

**Supplemental Methods and Analysis:**

**Phage-plasmid-like elements are found throughout diverse environments and encode niche-specific functional traits**

Mullet, J.I.^1,3^, Zhang, L.^2^, Pruden, A.,^1*^ Brown, C.L.^1*^

^1^Department of Civil and Environmental Engineering, Virginia Tech

^2^Department of Computer Science, Virginia Tech

^3^Department of Civil and Environmental Engineering, Massachusetts Institute of Technology

^*^co-corresponding authors

**Included in this PDF file:**

**Supplemental Methods:**

- S1 Text – Usage of mobileOG-db in PPLEs Prediction
- S2 Text – Analysis of RF Classifier
- S3 Text – Impacts of different clustering tools
- S4 Text – Additional accessory gene checks
- S5 Text – Analyzing VFs, MRGs, defense systems, and metabolism genes

**Supplementary Figures:**

- S1 Figure – mobileOG-db results for RF testing data
- S2 Figure – Confusion Matrix Results of RF Model
- S3 Figure – Amino Acid Identity (AAI) Comparison of identified PPLEs
- S4 Figure – Density Plot of Genome Sizes between MGEs
- S5 Figure – Density Plot of Genome Sizes between PPLEs
- S6 Figure – Taxonomic Classifications of PPLEs
- S7 Figure – Plasmidfinder results of PPLEs
- S8 Figure – MobMess PPLE clustering
- S9 Figure – Host Range Backbone Analysis
- S10 Figure – ARG Composition Analysis between MGEs
- S11 Figure – Visualization of PPLEs containing a CRISPR-Cas and anti-CRISPR system
- S12 Figure – Correlation Analysis between PPLEs accessory genes
- S13 Figure – Metabolic Analysis of PPLEs
- S14 Figure – Analysis of dTDP-6-deoxy-α-D-allose biosynthesis pathway
- S15 Figure – Comparison between MMseqs2 and CD-HIT clustering
- S16 Figure – Analysis of ARG detection cutoffs
- S17 Figure – Defense System Analysis
- S18 Figure – VF and MRG Analysis
- S19 Figure – tyPPing Comparison
- S20 Figure – IMG/VR Completeness Distribution
- S21 Figure – IMG/VR Quality Distribution

**S1 Text. Analysis of the mobileOG-db in the prediction of phage-plasmid-like elements**

Previous works classified phage-plasmids using HMMs and obtained weighted gene repertoire relatedness (1). However, we wanted to determine whether using mobileOG-db would improve the ability to classify phage-plasmids from a wider array of environments and with metagenomic sequences (2). The mobileOG-db database incorporates proteins from a wide array of mobile genetic elements, including phages, plasmids, insertion sequences, and integrative and conjugative elements, among others (2). This database, therefore, offers the potential to classify phage-plasmids from complex metagenomes with better functional annotations of other MGE classes – something that was not previously a focus of past phage-plasmid work (2). The complex interactions of gene transfer between MGEs make studying these elements particularly difficult in metagenomic sequences. Therefore, using mobileOG-db allows for more thorough annotation of MGE proteins, allowing for a more expansive and accurate phage-plasmid identification in metagenomic data.

To analyze whether mobileOG-db would work as an effective tool, we first examined the distribution of phages, plasmids, and phage-plasmid-like element proteins classified using mobileOG-db (2). We found statistically significant differences in the distributions of protein identification between the three groups of elements (Fischer exact test; p < 0.001) (Figure 1 in S1 Supplementary Methods). These results demonstrated the potential use of this database in classifying phage-plasmid-like elements. After these preliminary results, we attempted to train an RF classifier as described in the methods of the paper.

**S2 Text. Analysis of the predicted phage-plasmid-like elements from the machine learning model**

To compare the distribution of phage-plasmid-like elements between this paper and the phage-plasmids identified in previous works, we compared the elements found in both studies (3). Of the 1,416 previously predicted phage-plasmids, 1,318 were present in the databases used for data mining in this paper. Therefore, a maximum of 1,318 phage-plasmids from the prior studies could be detected using our classifier. This discrepancy is likely due to the use of different databases mined for phage-plasmids. Notably, of the previous 1,416 characterized phage-plasmids, 1,208 of these elements were found in PLSDB v.2021_06_23_v2 used in this analysis (4). Pfeifer et al. did not directly use PLSDB in the analysis but instead utilized NCBI RefSeq (4). When we searched across these two databases, we found an excess of plasmids in the PLSDB database (n=34,513) compared to the NCBI RefSeq version used in the prior work (plasmids from PLSDB found in NCBI n=19,226). This analysis was performed by downloading all of the genomes from the Pfeifer et al. paper and using the Biopython Package. Therefore, the use of different databases explains the variation in the number and distribution of phage-plasmids between studies. Additionally, due to the classifier's versatility, this paper obtained phage-plasmids from IMG/VR, thereby increasing the number of known phage-plasmids used (5).

We trained our model on the 780 phage-plasmids identified in prior work, allowing us to examine the efficiency of our machine-learning model compared to prior methods (1). Comparing the number of identified phage-plasmids to previously identified elements provides a better understanding of the reproducibility of phage-plasmid detection and the limitations of our model. Of these phage-plasmids, we recovered 1,124 of 1,318 (85.3%) previously identified elements in our testing/training data. Of the total 1,416 P-Ps from PMC9600943, we detected 72.5% of the new P-Ps. Additionally, we classified 99.9% of the plasmids and 100% of the phages from PMC7969092 as corresponding negative classes. This result correlates well with the confusion matrix predicted precision and recall data. This conservative classifier model was designed to limit false positives from metagenomic sequences. Therefore, this model was used to identify all 5,712 phage-plasmid-like elements (PPLEs) used in this analysis.

             To further validate the model, we compared the P-Ps from tyPPing, a tool designed to type P-P sequences based on existing curated classes (30). This tool uses a selective database of HMMs to identify protein families of experimentally validated P-P groups (30). We found that only 1,055 of 5,712 PPLEs genomes detected in this study could be placed into these classes. Similarly, we found that less than half (601 of 1,416) P-Ps reported from PMC9600943 could be classified using tyPPing (31). While tyPPing demonstrates an effective method for classifying small, verified groups of P-Ps, there are still many novel and unknown groups of P-Ps that need to be validated and discovered.

            These analyses show that this model performs comparably to previous works (1). Some of the differences are likely due to differences in the hallmark proteins used to classify P-Ps. The use of mobileOG-db has the unique ability to identify proteins across a wide array of MGEs and provide functional descriptions for them. This approach provides more information and context that should result in a more conservative but confident set of phage-plasmid-like elements. That was the primary reason for implementing this method for detecting phage-plasmids from datasets containing metagenomes such as IMG/VR (5). However, additional work is needed to validate these PPLEs experimentally.

**S3 Text. Examining the impact of clustering on phage-plasmid-like elements (PPLEs)**

To analyze how clustering using different tools can impact the PPLEs, we compared our results to MMSeqs2 using a percent identity of 95% and query coverage of 85% (6). These thresholds represent a standard cutoff used in various phage metagenomic experiments (7). Clustering using CD-HIT and MMSeqs2 resulted in similar numbers of clustered PPLEs. MMSeqs2 resulted in 6,036 phage-plasmid-like clusters at 95% identity, and CD-HIT obtained 5,712 PPLE clusters at 97% identity (6,8). To compare how the different clustering tools impacted the distribution of similar PPLEs, we performed an all-by-all comparison of the ANI values using FastANI of all PPLE representatives obtained from CD-HIT and MMseqs2 (6, 8, 9). A genome density plot was created that contained all ANI values (>80% ANI) for the pairwise comparison of all the PPLEs obtained with each tool (9). This analysis demonstrates that both clustering tools result in similar PPLE ANI distributions (Figure 15 in S1 Supplementary Methods). We noticed a couple of interesting results with this comparison. The clustering tools performed relatively similarly, but MMSeqs2 at a 95% identity did result in more overall clusters (6). Additionally, both tools are imperfect, and some genomes remained above the percent identity threshold for both tools (Figure 15 in S1 Supplementary Methods).

To examine whether these different clustering tools altered any significant findings within this paper, we compared the anti-CRISPR genes that were analyzed before using the same procedure mentioned in the methods and materials. We performed a Fischer exact test comparing the clustered PPLEs using both MMSeqs2 and CD-HIT with the phage and plasmid test datasets. The anti-CRISPR genes were still significantly different in PPLEs when using both tools compared to phages and plasmids (Fischer exact test; 267 PPLEs genes vs. 5 phage genes vs 1 plasmid genes; p < 0.001). These results indicate that while the number of clusters is different, the overall trends and general usage from both CD-HIT and MMSeqs2 both work for this study (6, 8). We chose to utilize the CD-HIT clustering for the main text paper, but the accession IDs for the unclustered and MMseqs2 clustered PPLEs obtained in this study are available on the github (6, 8).

**S4 Text. Accessory Gene Analysis**

We examined the cutoffs used and the correlation between genes to examine further the effectiveness of the accessory genes identified in this study. An 80% percent identity cutoff was used to identify several classes of accessory genes, such as antibiotic resistance genes. While this is a common cutoff used in multiple analyses, we wanted to examine whether this was an appropriate cutoff to use in this analysis, particularly how this cutoff affected the data (10). We obtained the various percent identity values for all antibiotic resistance genes classified using our blast results. The results showed that less than 2% of all ARGs classified in this paper had a percent identity of less than 90%, which improves the confidence in the use of this threshold for ARGs (Figure 16 in S1 Supplementary Methods).

Previous work has examined the correlations between various accessory gene content in MGEs. In particular, research has examined potential inverse relationships between the carriage of defense systems and the carriage of antimicrobial resistance and virulence factor accessory genes (11). We wanted to examine the correlation of these accessory genes in PPLEs to determine whether any similar trends exist. Looking across all of the accessory genes identified in this paper, we found positive correlations across most of the accessory genes throughout most environments (Figure 12 in S1 Supplementary Methods). Generally, PPLEs with more accessory genes of one class contain more flexible genes across all classes.

**S5 Text. Comparing the diversity and gene content of PPLE** **accessory genes for defense systems, metabolism genes, metal resistance genes, and virulence factors**

To further survey and investigate the various accessory genes found within PPLEs, we dug into some other categories of accessory genes. To do this, we compared different accessory genes such as metabolism genes, virulence factors, defense systems, and metal resistance genes. Examining these genes in different environments can elucidate a more biological understanding of how these elements interact within their environments. To ensure that the increased average size of PPLEs did not explain the enrichment of certain accessory genes in phage-plasmid-like elements, we normalized the data to the genome size. We analyzed anti-CRISPR genes to observe whether these trends remain consistent. We analyzed the frequency of anti-CRISPR genes compared to the combined number of genes from the classified phage, plasmid, and PPLEs classes and found accessory genes such as anti-CRISPR genes were still enriched when accounting for differences in genome size (Fischer Exact Test with a Benjamini-Hochberg correction; p < 0.001).

First, we examined the unique distribution of defense systems found in phage-plasmids-like elements. When examining the defense systems, it is first interesting to note that PPLEs can possess a diverse assortment of defense systems across environments (Figure 17 in S1 Supplementary Methods). Restriction Modification systems dominated the most common defense systems found in PPLEs across all environments (Figure 17 in S1 Supplementary Methods). CRISPR-Cas systems were the next most frequent defense system (Figure 16 in S1 Supplementary Methods). We decided to examine these and the potential of anti-CRISPR genes in the main text. Some defense systems were found in uniquely host-associated PPLEs (DPD, DRT, Haichiman, Kiwa, and PBE), while others were found across most environments. There were a few, such as GAO 19 and GAO 20 systems, only found in wastewater-sourced PPLEs, and some, like qatABCD, only found in terrestrial PPLEs. The diversity of both the distribution and diversity of defense systems found in PPLEs demonstrates the diversity of these elements.

We then examined the metabolic modules found across environments, which was done with the assistance of Microbeannotator (12). Pyrimidine metabolism, methionine degradation, nicotine degradation, and tetrafolate biosynthesis were the most abundant metabolic genes found in PPLEs. We further examined specific metabolic pathways to compare these genes across environments (Figure 13 in S1 Supplementary Methods). Several pathways, such as biosynthesis of secondary metabolites, pyrimidine metabolism, and nitrogen metabolism, were found across most environments. We found polyketide sugar unit biosynthesis particularly interesting and warranted further analysis. It was the most complete metabolic gene cluster and appeared enriched in the aquatic PPLEs only. These differences show that there are some metabolic gene-carrying differences. However, PPLEs across an array of environments have the potential to hold a diverse arsenal of metabolic genes.

To further examine the diversity of the metabolic accessory genes found on PPLEs, we considered the dTDP-6-deoxy-α-D-allose biosynthesis pathway (Figure 14 in S1 Supplementary MethodsThis pathway is critical for the formation of mycinose, as dTDP-6-deoxy-α-D-allose is the last free intermediate in this biosynthesis pathway (13). Mycinose is an important biomolecule that assists in forming several macrolide antibiotics (13). The PPLEs that possess this metabolic pathway were exclusively aquatic PPLEs, and all contained identical KEGG Modules (M00794) (14). In particular, these aquatic PPLEs contained three of the four enzymes in this pathway, including dTDP glucose 4,6-dehydratase, an enzyme that assists in forming all 6-deoxy sugar biosynthesis (Figure 18 in S1 Supplementary Methods)) (15). These PPLEs contained genes encoding two enzymes (dTDP-4-dehydro-6-deoxy-D-glucose-3-epimerase and dTDP-4-dehydro-6-deoxy-α-D-gulose-4-ketoreducatase) that are essential to the dTDP-6-deoxy-α-D-allose biosynthesis pathway (16). The presence of the intermediate steps of the nucleotide sugar pathways (e.g., Figure 14 in S1 Supplementary Methods) suggests that PPLEs could stimulate auxiliary metabolite production from host-derived inputs of glucose 1-phosphate, dTTPs, and thymidyltransferase. Many polyketide sugars are frequently associated precursors for bacterial-produced antibiotic pathways, and these were exclusively found in aquatic PPLEs.

To further investigate accessory gene trends, we also examined both MRGs and virulence factors. Both groups of flexible genes were predominantly found in PPLEs from host-associated or wastewater environments. For MRGs, mercury, arsenic, silver, and biocide resistance were the most commonly found genes (Figure 18 in S1 Supplementary Methods)). For virulence factor genes, the most frequent categories were immune modulation, effector delivery systems, and adherence (Figure 18 in S1 Supplementary Methods)). These overall trends were consistent with the trends with defense systems and ARGs.

Supplemental Figures:

**
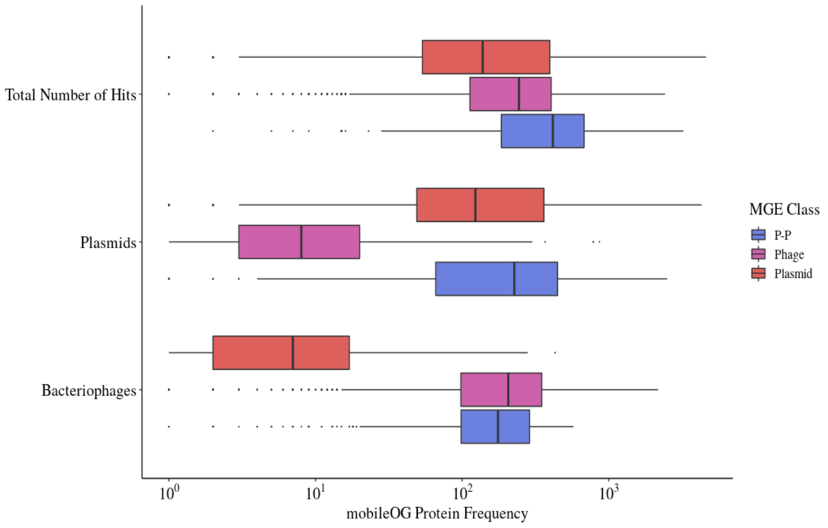
**

**Supplemental Figure 1** – Breakdown of the mobileOG-db protein hits for the testing data from Pfeifer et al. 2021 examining the phage, plasmid, and total protein hits for each of the major classes (1, 2). This Figure displays the total number of proteins from the phage, plasmid, and total hits from the Major Categories in mobileOG-db.

**
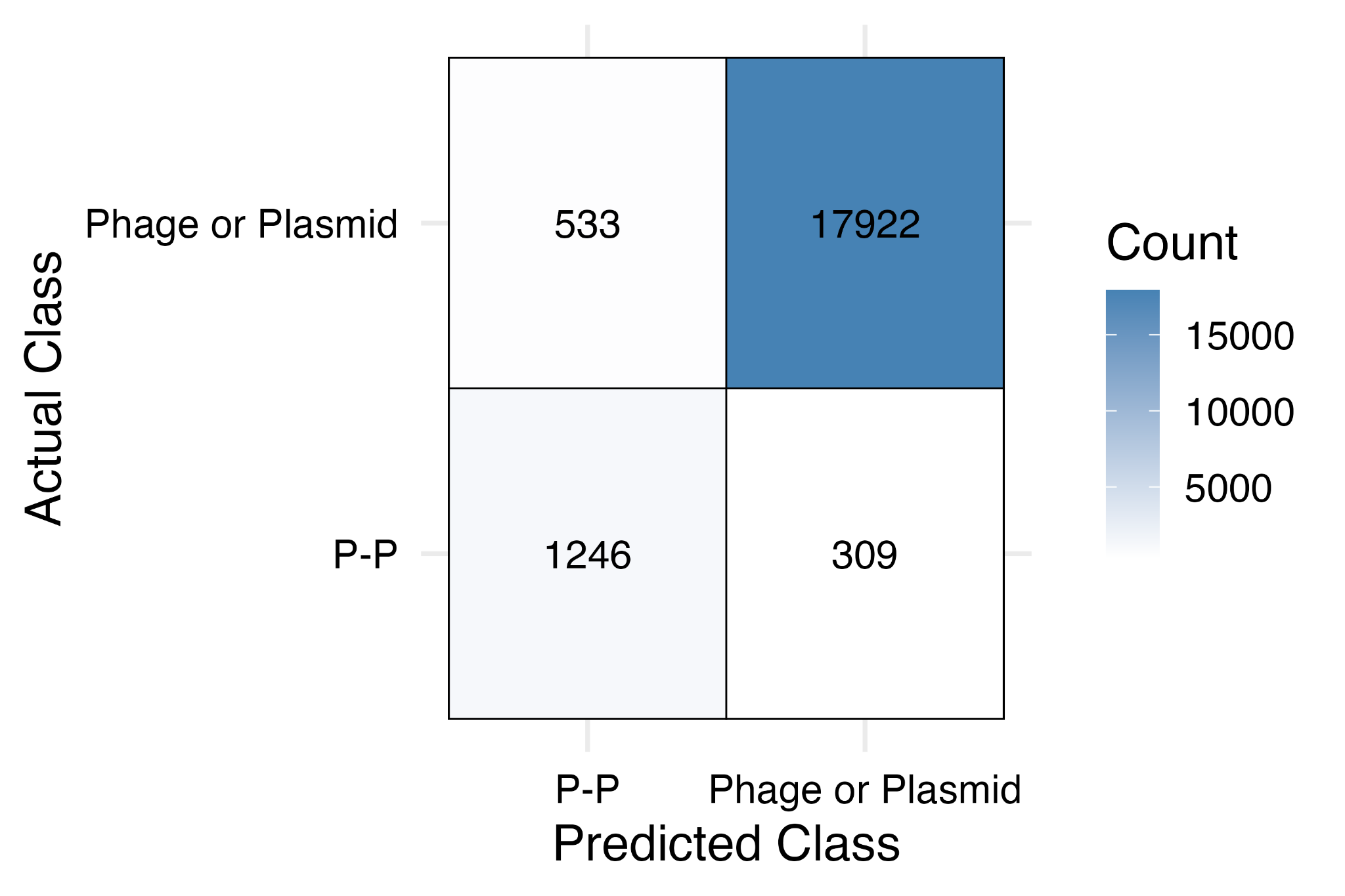
**

**Supplemental Figure 2** – A confusion matrix containing the sum of the 10 randomized training sets used to validate the model performance. The details of each randomized model is available in the supplementary table 1.


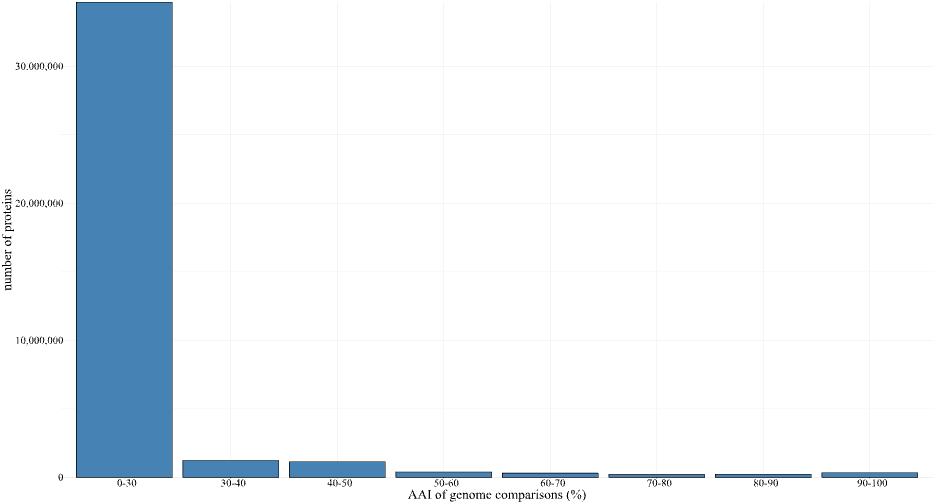


**Supplemental Figure 3** – Histogram plot depicting the distribution of AAI values among all of the identified PPLEs used in this experiment. AAI values were calculated using the CompareM tool for an all-by-all comparison of respective genomes (17).


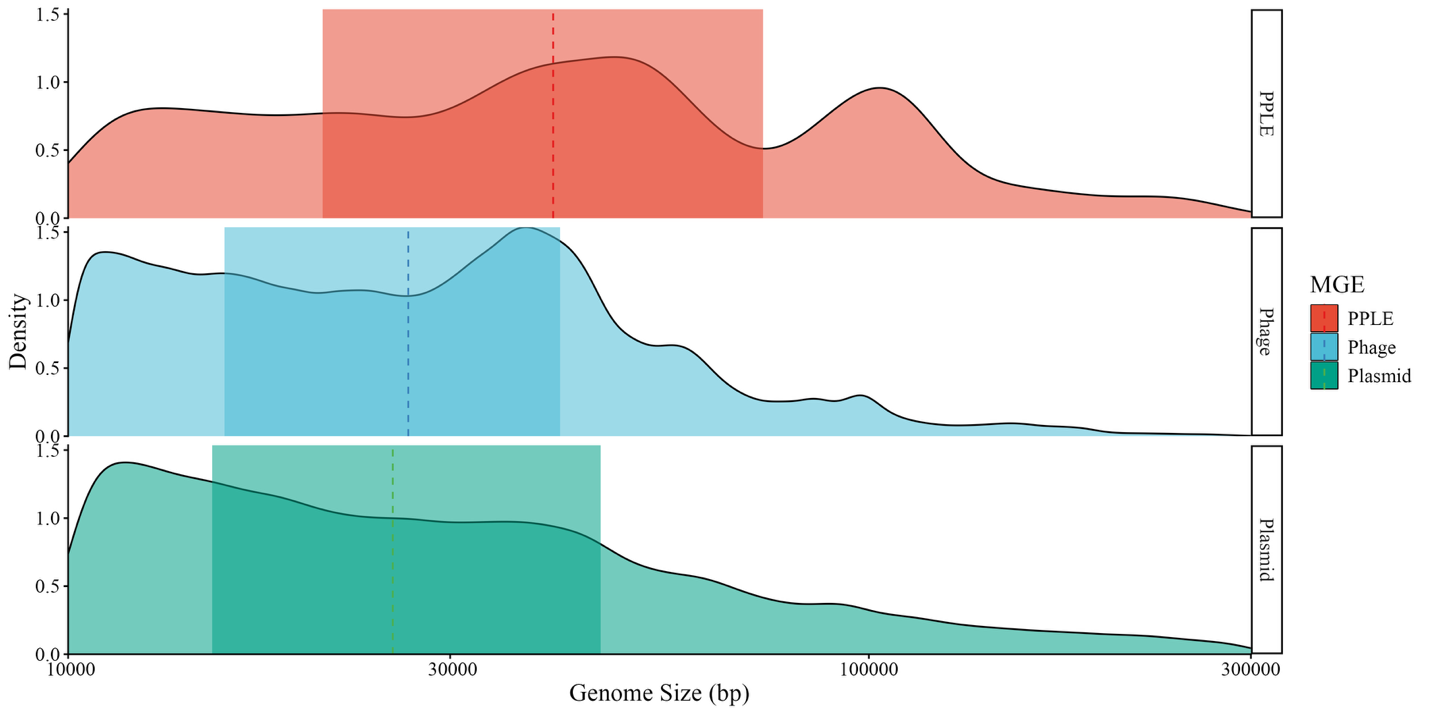


**Supplemental Figure 4** – Density plot of the genome size distribution of phages, plasmids, and PPLEs. The boxes represent the boundaries of the 1st and 3rd quartiles of the data while the dashed line shows the median genome size for each MGE type.


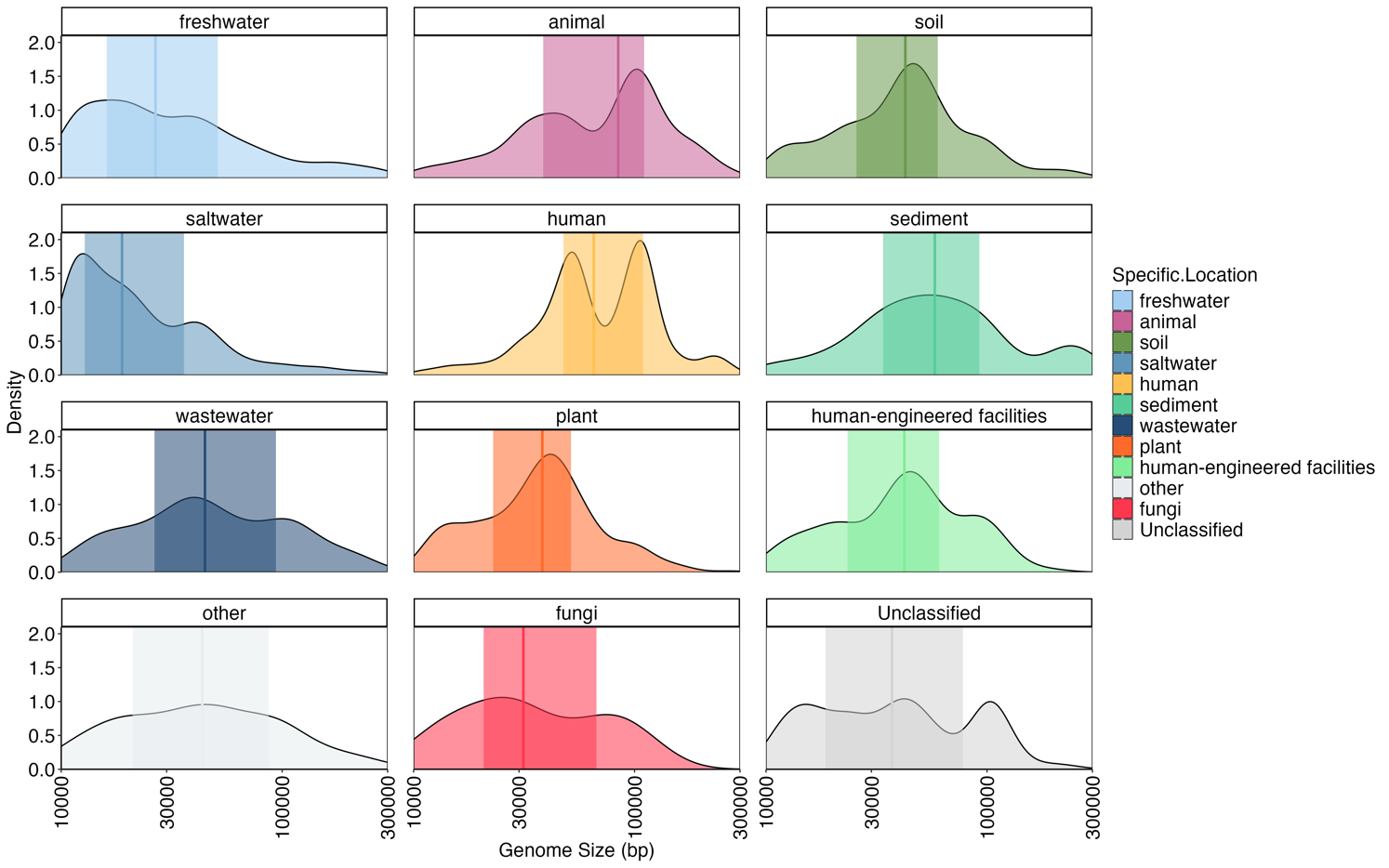


**Supplemental Figure 5** – Density plot of the genome size distribution of PPLEs across different environments. The boxes represent the boundaries of the 1st and 3rd quartiles of the data while the dashed line shows the median genome size for all PPLEs found from each environment.


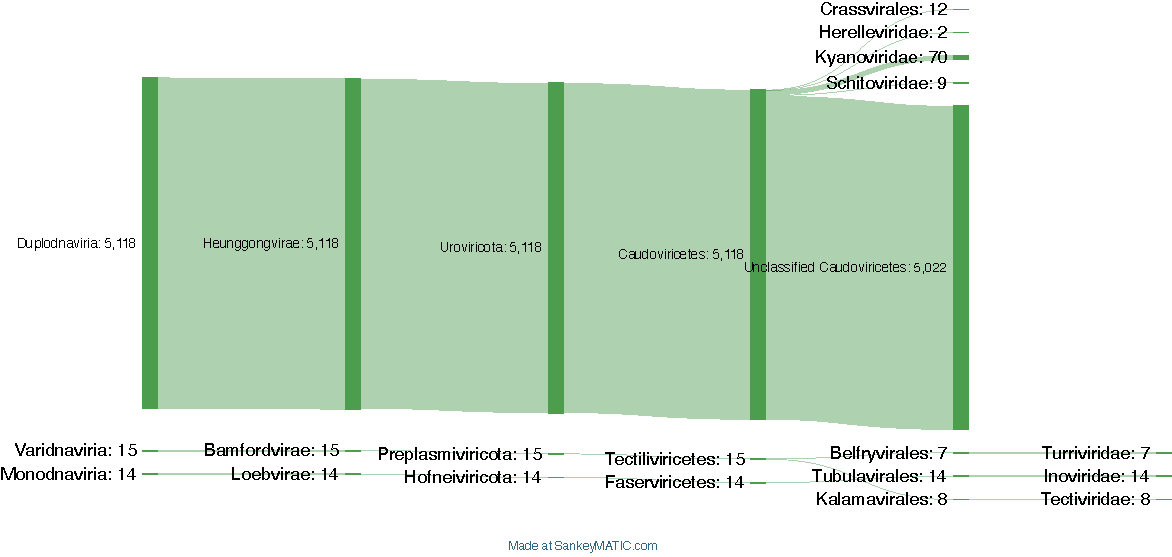


**Supplemental Figure 6** – Sankey plot of the viral taxonomic distribution of different phage-plasmids found in this experiment (18). The taxonomic classifications for each PPLE were determined using geNomad (19), and any infrequent taxonomic groups (n < 1) were removed from the above figure.


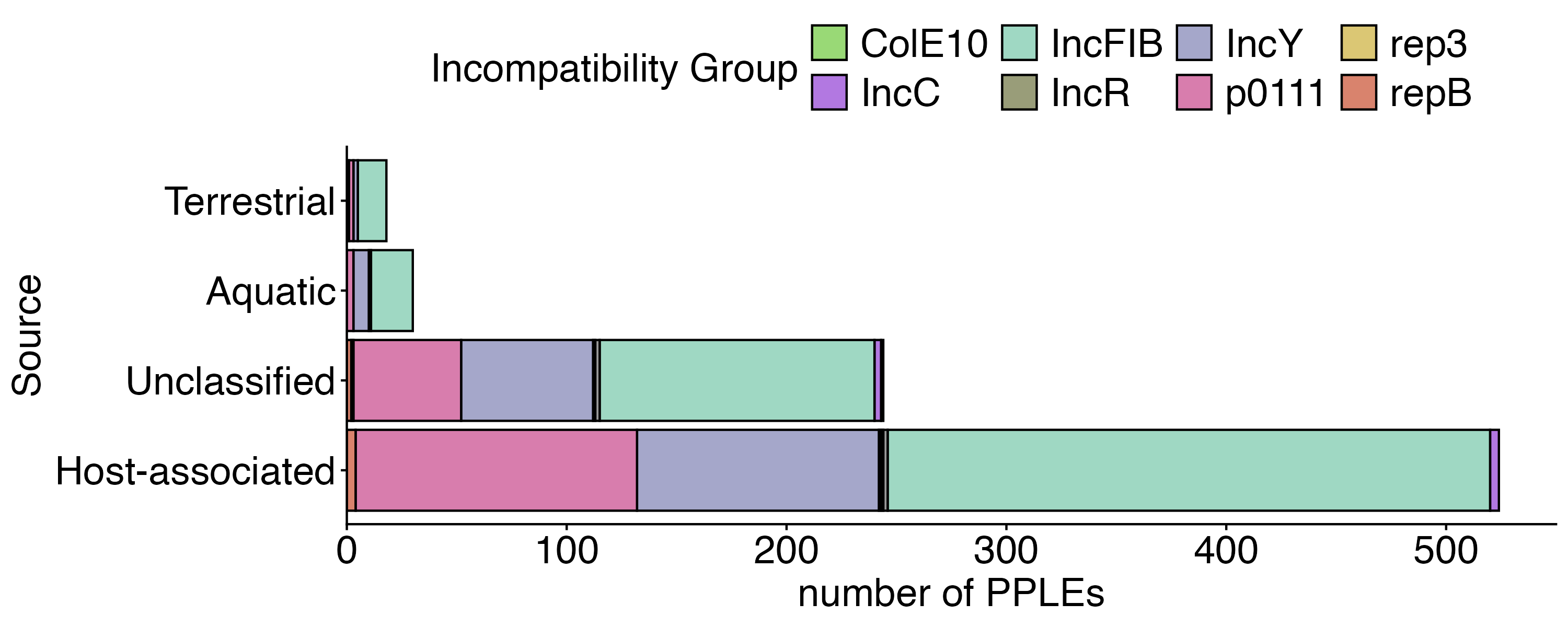


**Supplemental Figure 7** – Distribution of plasmid incompatibility groups from different environmental source locations. The incompatibility groups were determined using plasmidfinder v2.1.6, and any rare groups were removed from the above figure (20). Unclassified PPLEs denote any element that did not have a classified source location.


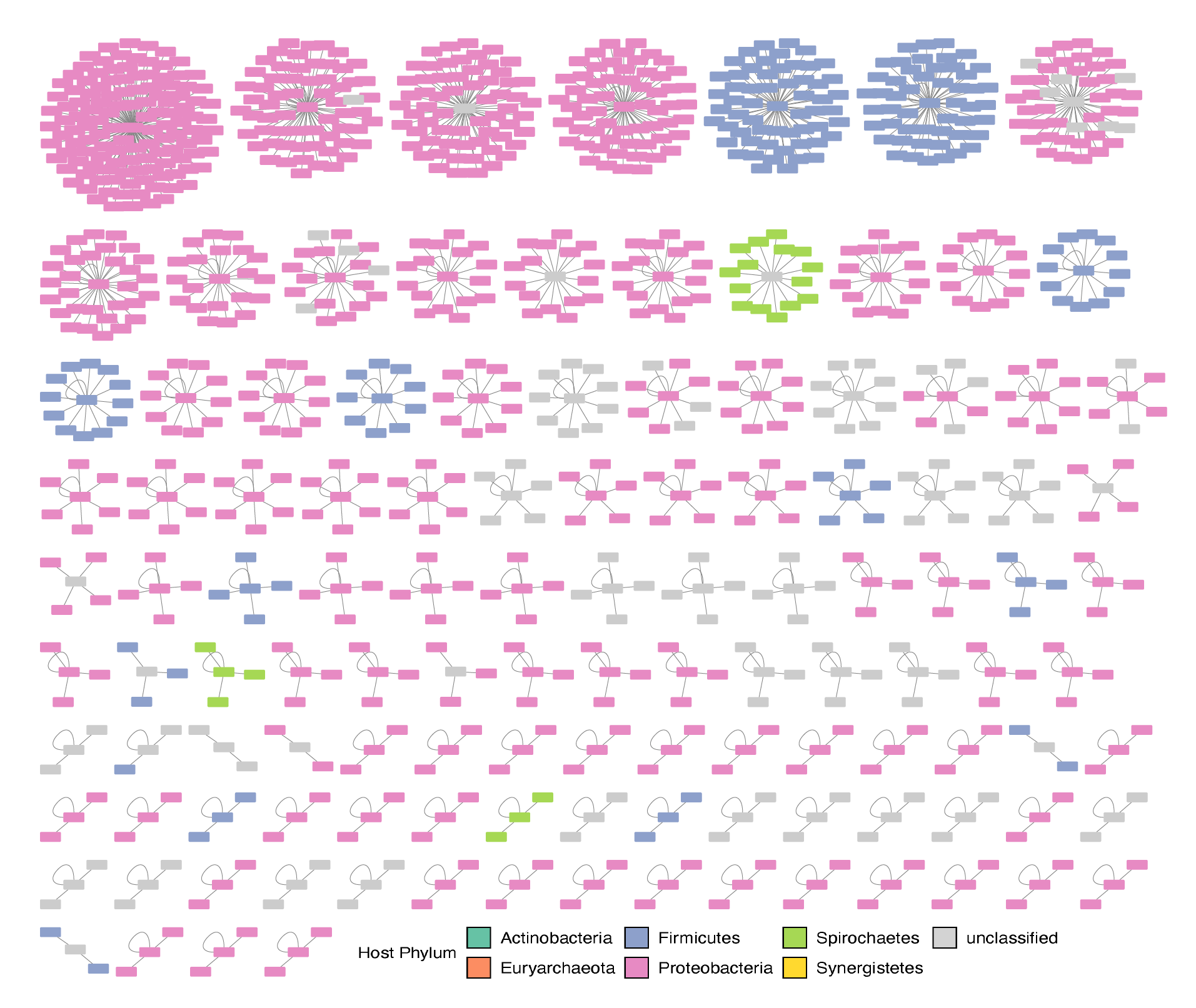


**Supplemental Figure 8** – Analysis of identified plasmid systems in PPLEs. Depicts the plasmid clustering results identified via MOBMess and Cytoscape (21, 22). Phage-plasmid-like elements with less than 3 PPLEs in a cluster were removed, and the predicted host taxonomy was overlaid onto all of the elements.


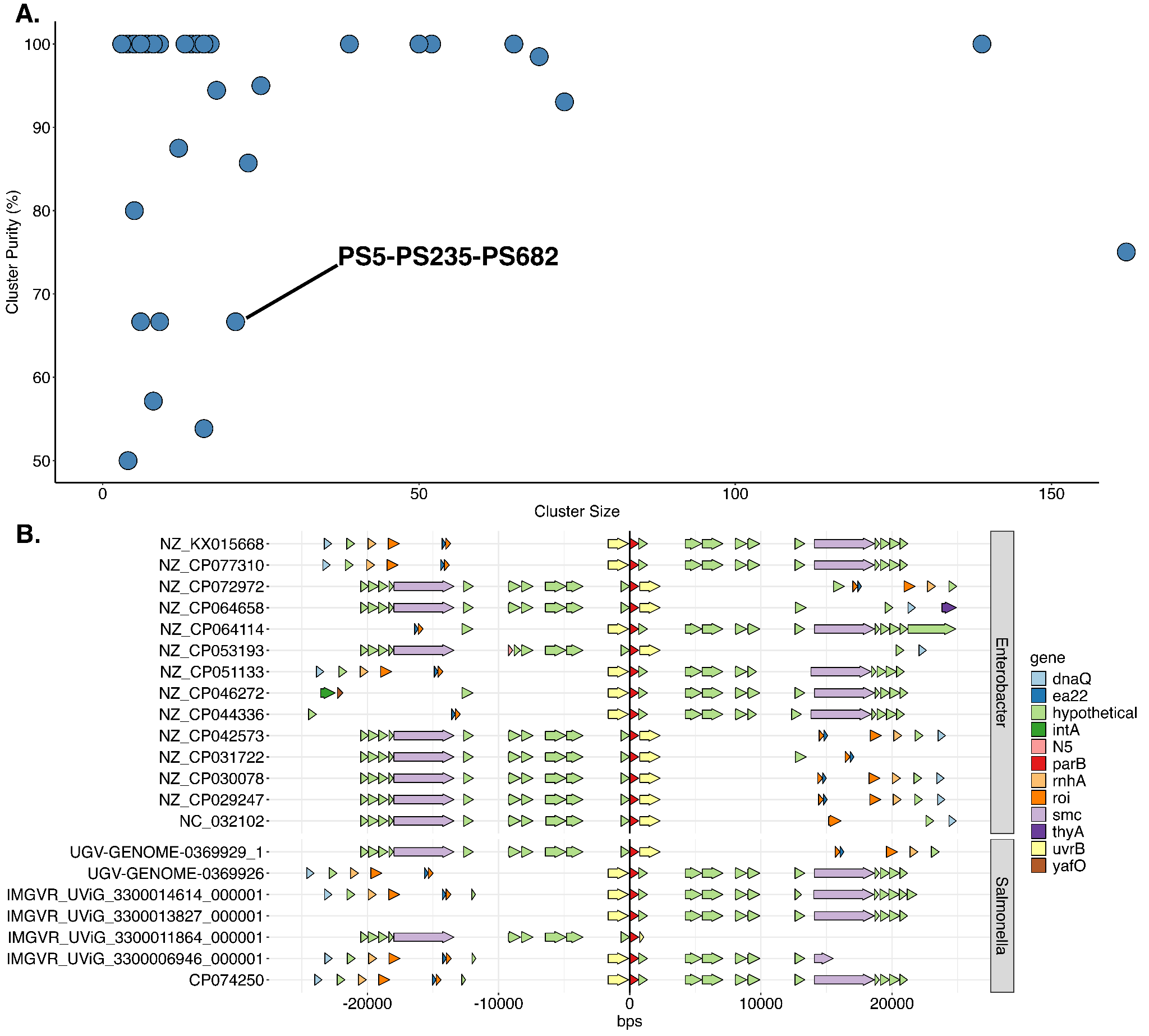


**Supplemental Figure 9** – Analysis of plasmid backbones of PPLEs. (A) Examining the cluster purity of plasmid backbone clusters at the genus level (21). Genomes with unclassified genus-level hosts were removed from the purity calculations. (B) Gene Alignment of the PPLEs containing the PS5|PS235|PS682 plasmid backbone using the gggenes package. Depicts gene-to-gene alignment of the parB gene grouped by respective host associated genus annotated by mobileOG-db (2). Only genes found in >1 genome and within 25,000 base pairs upstream or downstream of the parB gene are displayed.


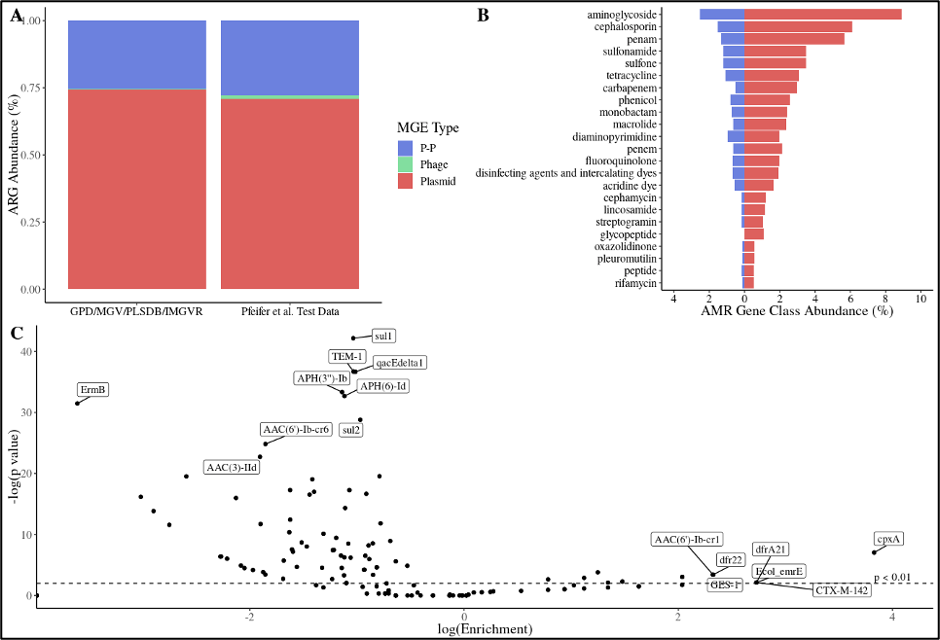


**Supplemental Figure 10** – ARG Composition Analysis between phages, plasmids, and phage-plasmid-like elements. (A) Depicts the relative abundance of ARGs in the respective MGE classes to account for differences in sampling. (B) Examines the unique AMR gene classes between plasmids and phage-plasmid-like elements. (C) Examines the statistical enrichment of unique ARGs in plasmids and phage-plasmid-like elements with genes enriched in plasmids appearing on the left and enriched phage-plasmid-like elements genes appearing on the right. The statistical enrichment and p-value were calculated using a Fischer Exact Test. The PPLE color represents PPLEs in this figure.

**
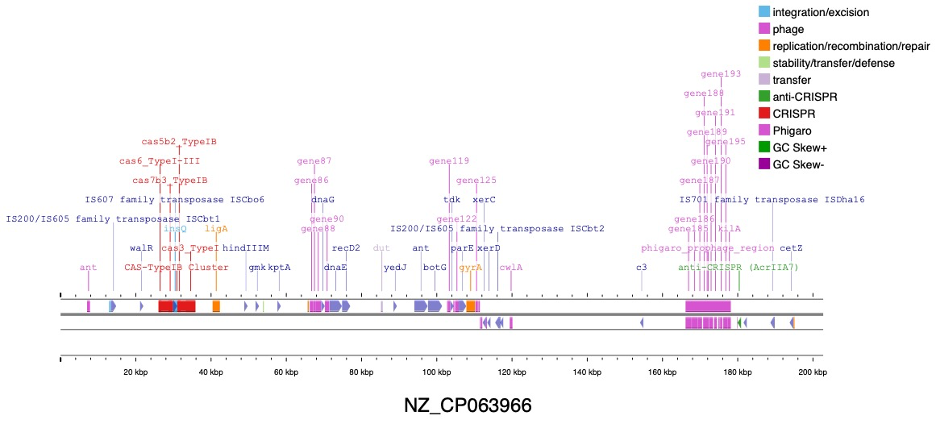
**

**Supplemental Figure 11** – Visualization of the phage-plasmid-like element containing both a CRISPR-Cas system and an anti-CRISPR gene region (NZ_CP063966). The element was visualized on Proksee using the Prokka, mobileOG-db, CRISPRCasFinder, and Phigaro functions (2, 23, 24, 25, 26).


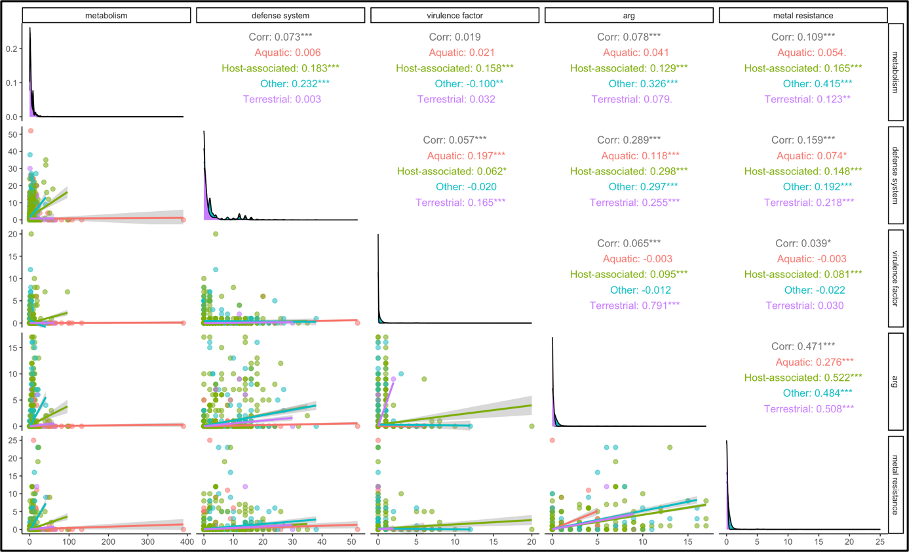


**Supplemental Figure 12** – Correlation plot between the various phage-plasmid-like element accessory genes. Anti-CRISPR and toxin-antitoxin systems were grouped into defense systems. The Figure was produced utilizing the ggpairs function associated with the GGally package in R. The statistical significance between the different correlation values are labeled (* = p < 0.05; ** = p < 0.01; *** = p < 0.001). All genomes designated as “Other” are phage-plasmid-like elements with unclassified source locations.


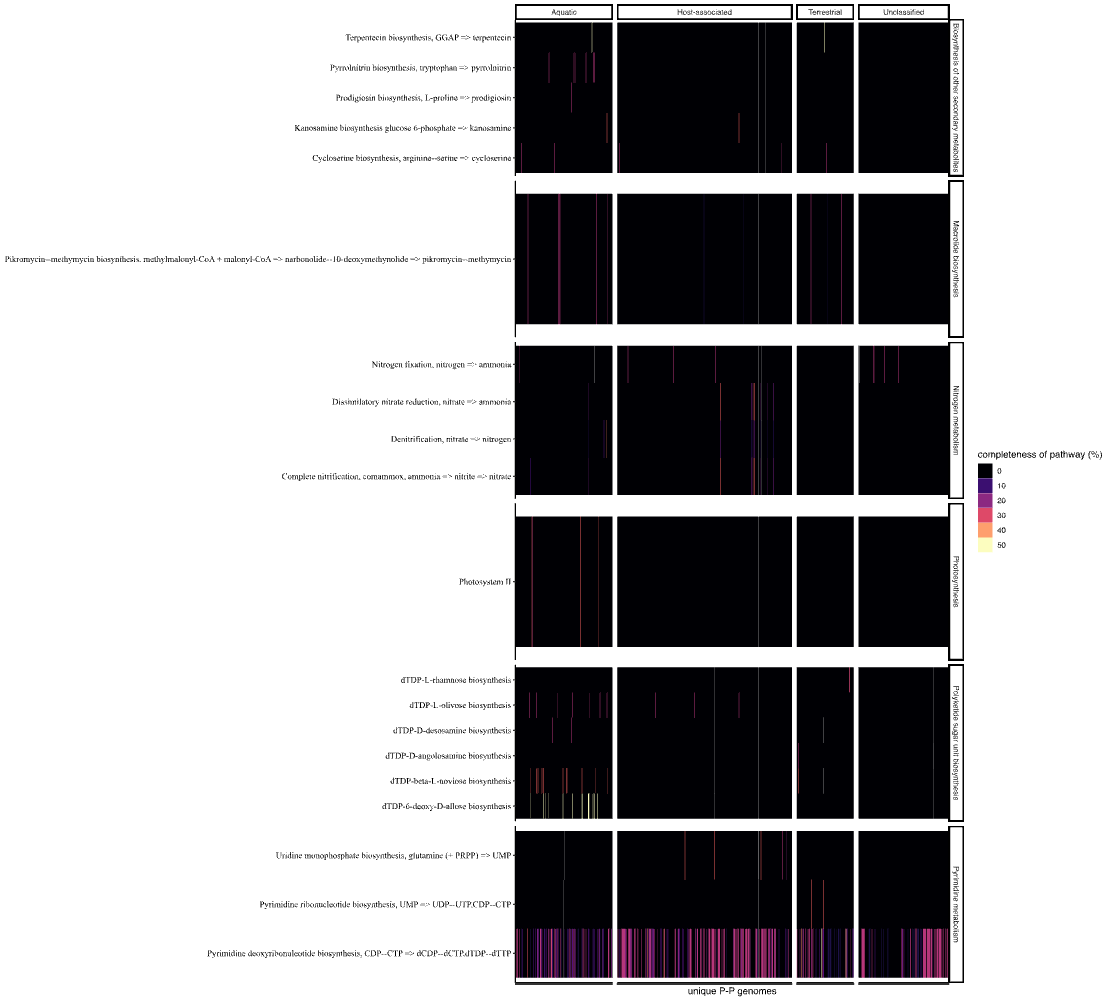


**Supplemental Figure 13** – Summary of the pathway completeness of key metabolic pathways found in different source location phage-plasmid-like elements from Microbeannotator (12). Only unique PPLE genomes with more than 1 metabolic pathway are shown in this plot.


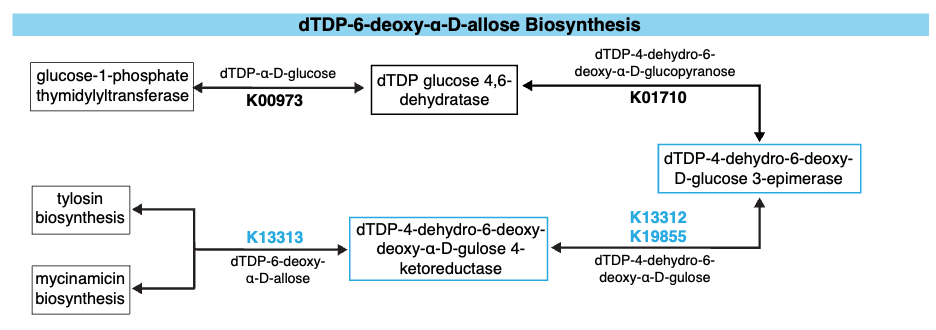


**Supplemental Figure 14** - dTDP-6-deoxy-α-D-allose biosynthesis pathway found in some aquatic PPLEs (n=14/1,868) (13, 14, 16). The blue-outlined boxes indicate the portion of the associated pathway found in the PPLEs. Among the 14 PPLEs found to carry portions of this pathway, 13 were derived from freshwater and one from saltwater. The designated KEGG pathways align with the reaction products from these enzymes with the blue KEGG pathways indicating portions of the pathway that the PPLE carried accessory genes (15).


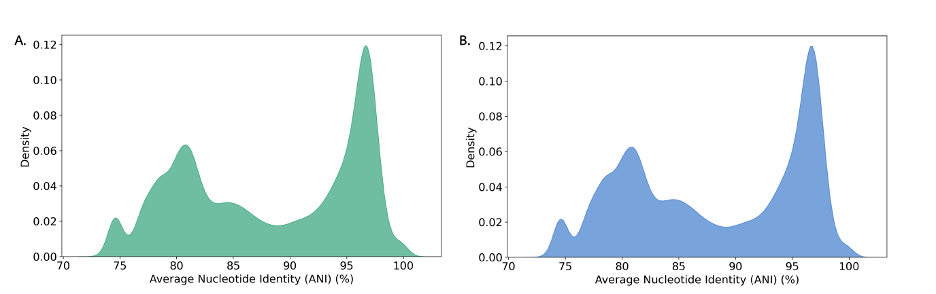


**Supplemental Figure 15** – Density Plot of the ANI of phage-plasmid-like elements using MMseqs2 and CD-HIT (6, 8). (A) The distribution of phage-plasmid-like elements using CD-HIT clustering at 97% identity (8). (B) The distribution of phage-plasmid-like elements using MMseqs2 clustering at 95% identity (6). The ANI values for the all-by-all comparison were performed using fastANI (9). The density plot only contains comparisons that contain ANI above 80% and are not representative of all phage-plasmid-like elements.


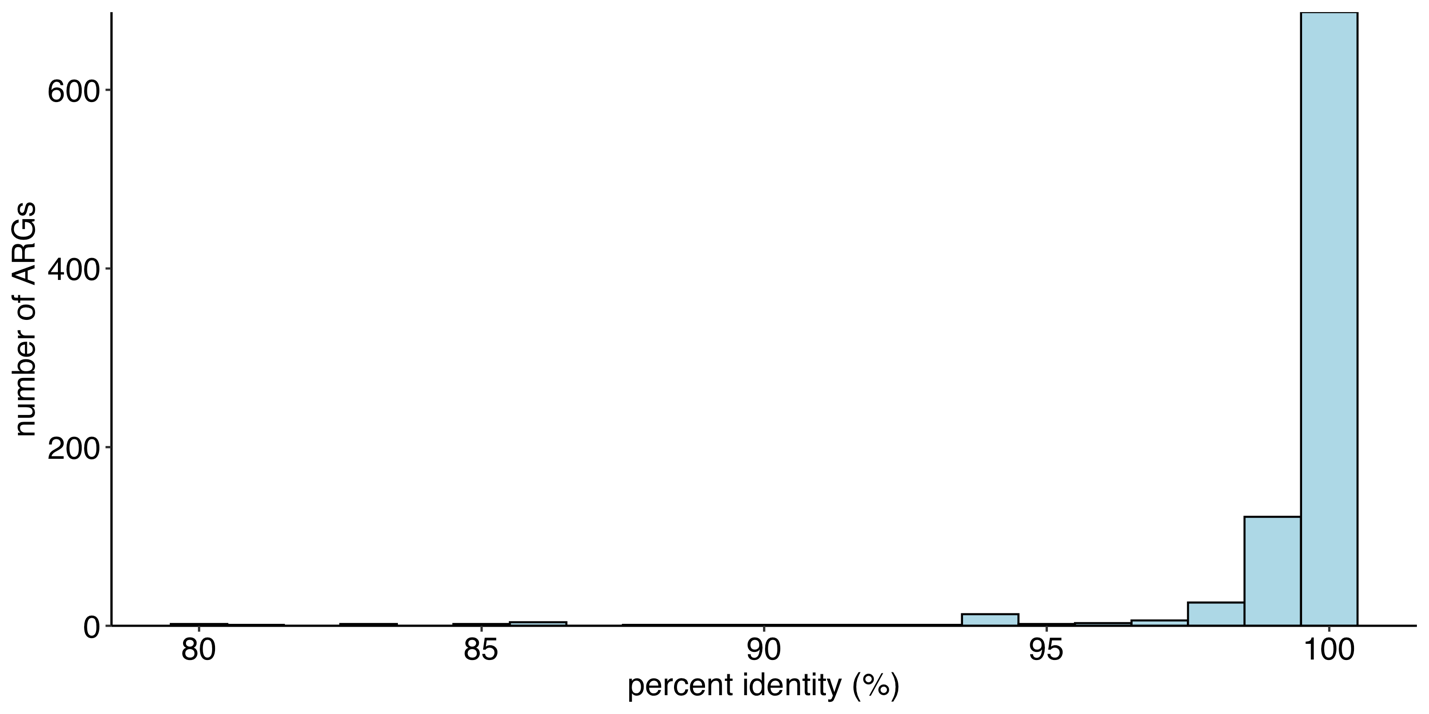


**Supplemental Figure 16** – Analysis of antibiotic resistance gene percent identity using BLAST. These results compare the percent identity of each ARG identified in this paper with the cutoff of 80% used in the workflow.


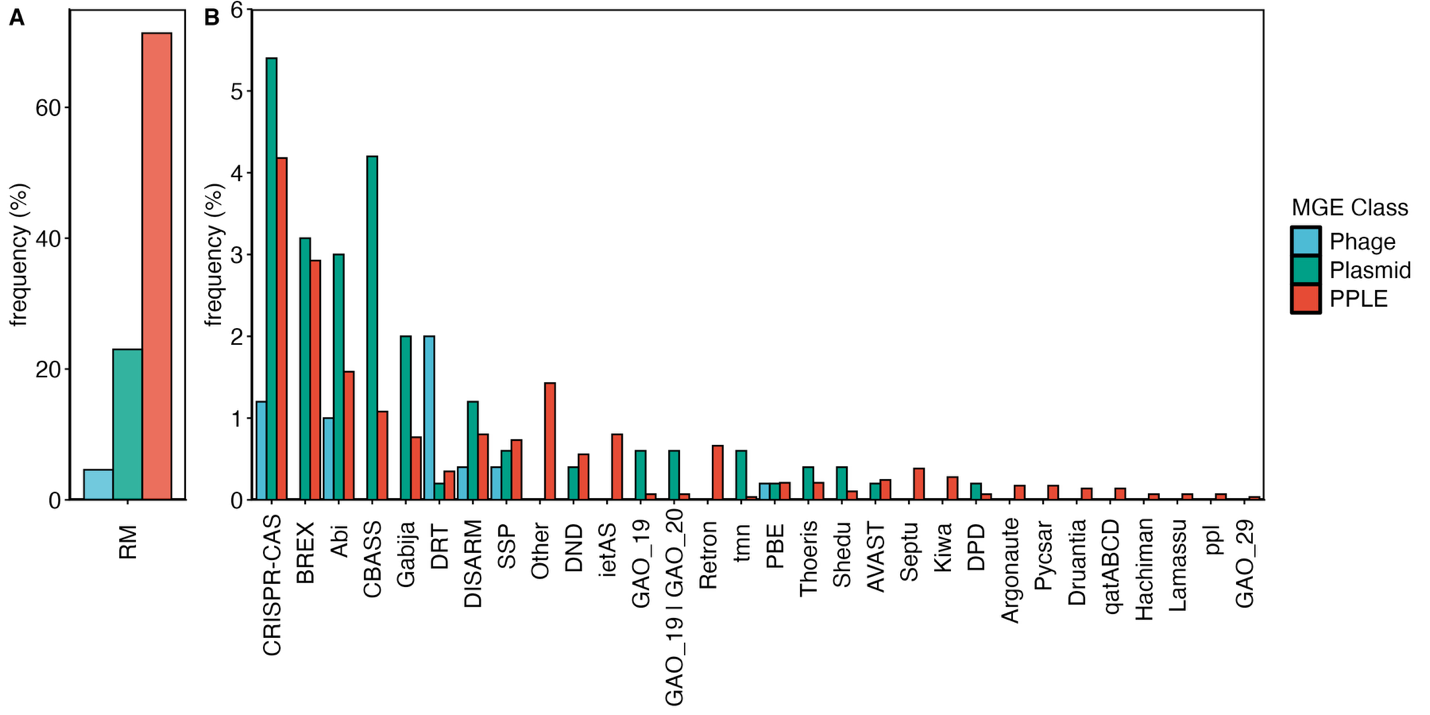


**Supplemental Figure 17** – Distribution of defense system genes detected in phage-plasmid-like elements across different environments. The gene counts were visualized on a log10 scale to better examine the less abundant defense system genes. All defense system genes were grouped by the generalized defense systems defined using PADLOC (27).


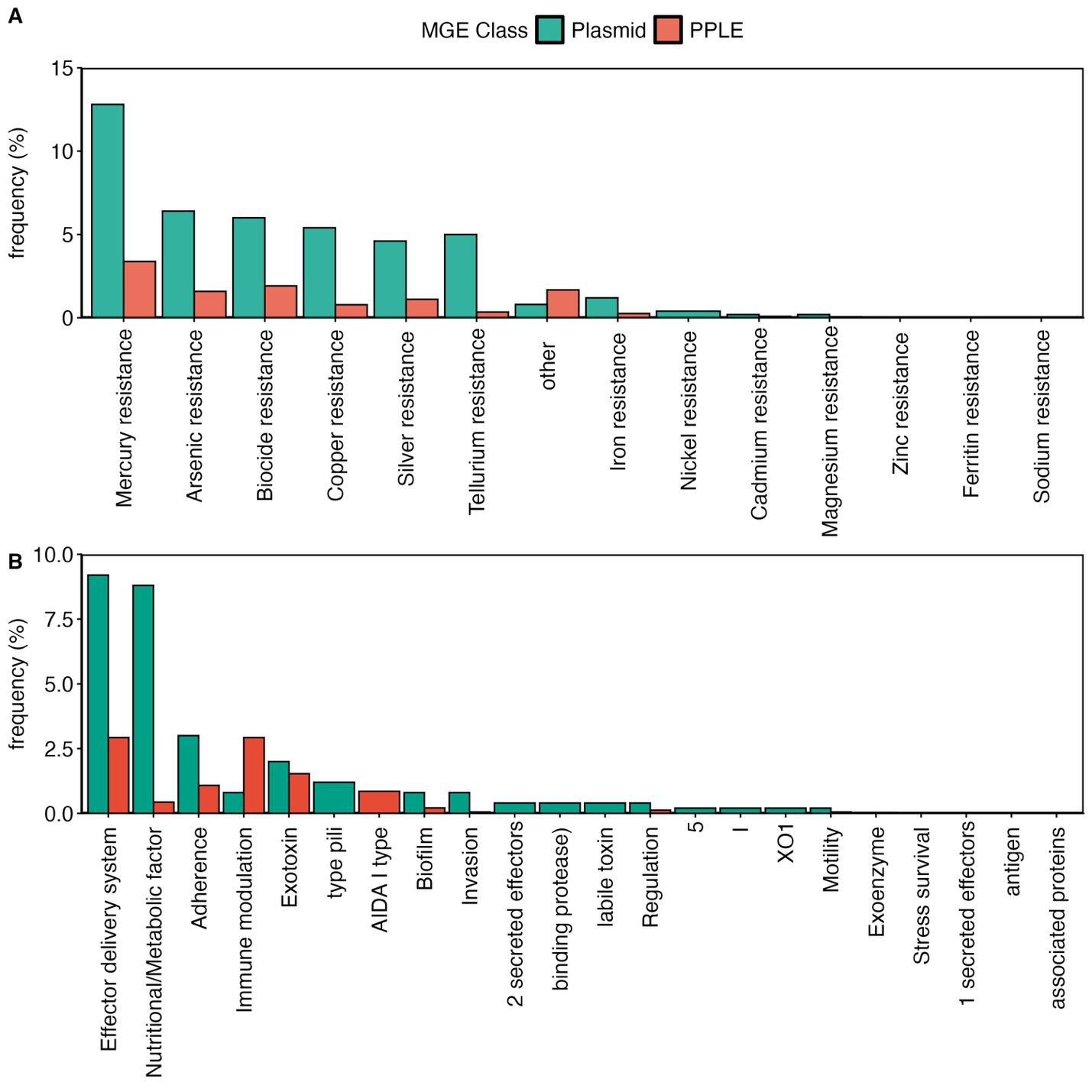


**Supplemental Figure 18** – (A) Relative frequency of metal resistance genes detected in phage-plasmid-like elements (PPLEs), plasmids, and phages. These were defined by the primary resistance groups as described by BacMet2 (28). (B) Relative frequency of virulence factors detected in phage-plasmid-like elements, plasmids, and phages. These were defined by the primary broad virulence factory categories defined using VFDB (29). Only the randomly subsetted 500 phages and plasmids were used in this analysis.


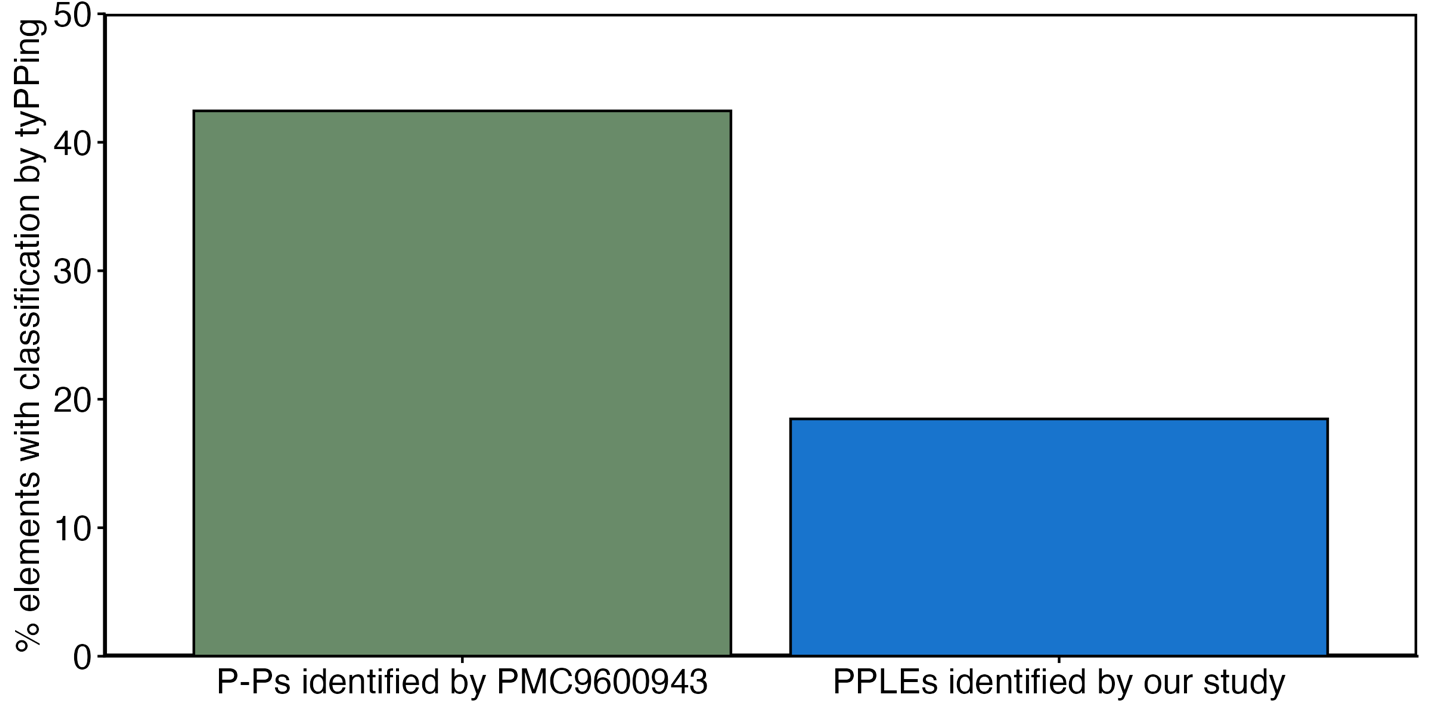


**Supplemental Figure 19** – The percentage of PPLEs with a classification using the tyPPing tool with default settings (30). The plot compares all the PPLEs identified from this study and the 1,416 PPLEs identified from Pfeifer et al. (PMC9600943) (30, 31).


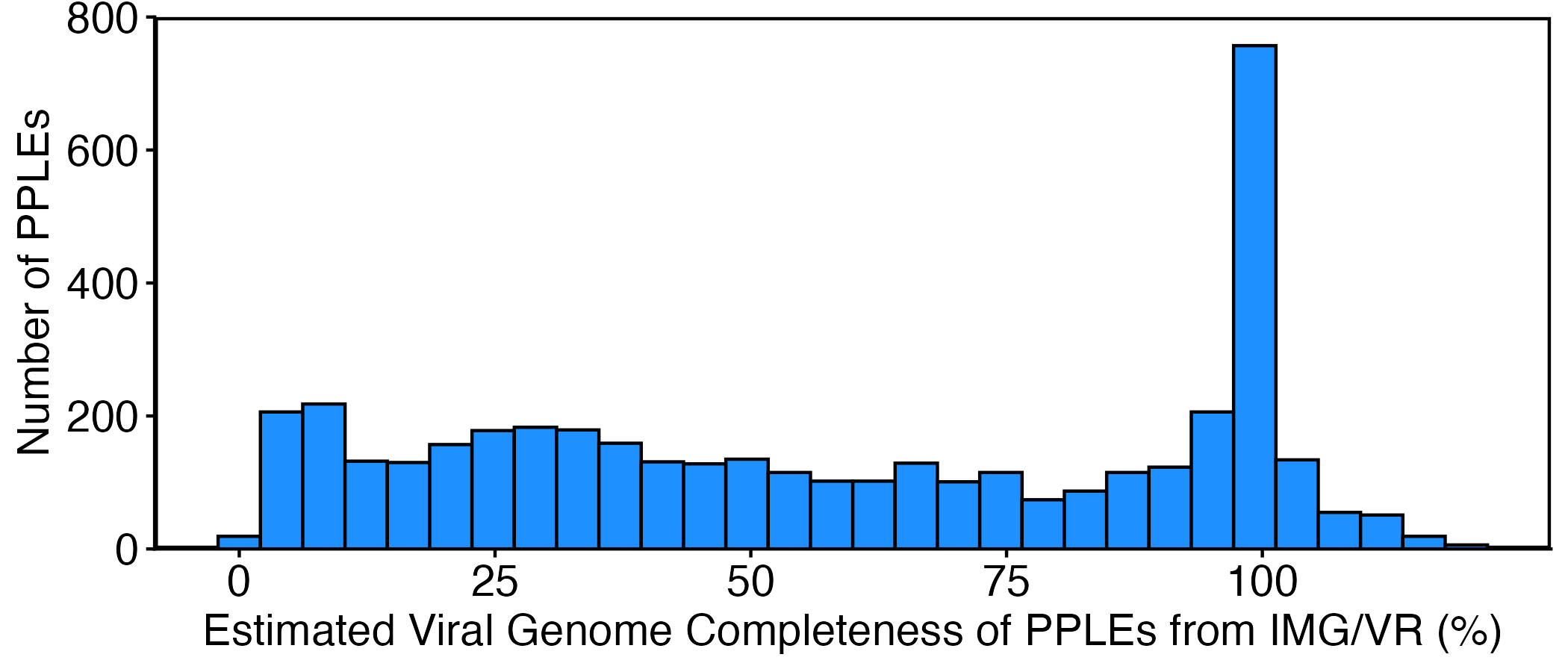


**Supplemental Figure 20** – The distribution of PPLEs based on estimated completeness of elements sourced from IMG/VR. The completeness was based on the metadata from IMG/VR.


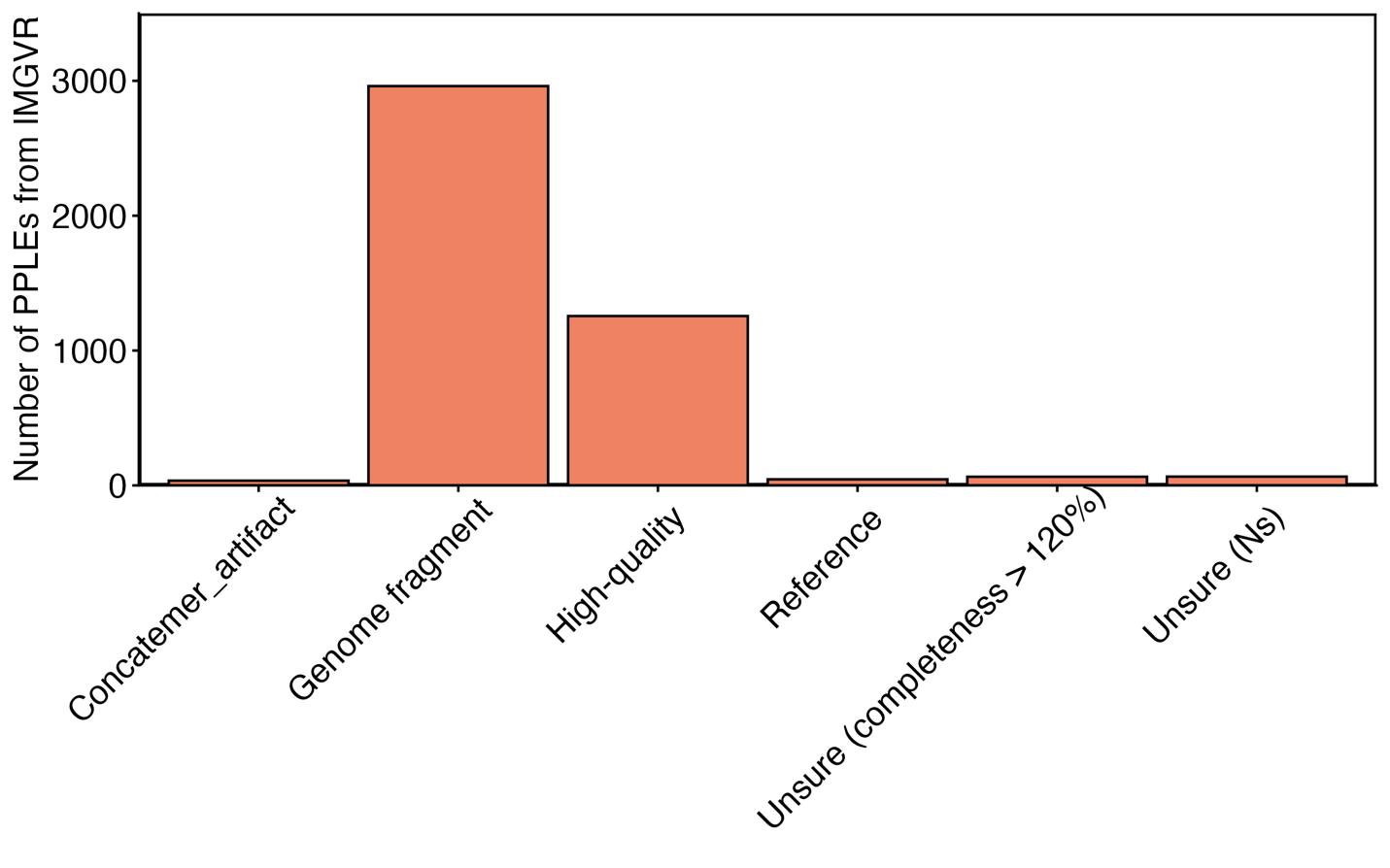


**Supplemental Figure 21** – The MIUViG quality of the PPLEs identified from IMG/VR. The quality scores used IMG/VR provided metadata.

**References:**

1. Pfeifer E, Moura De Sousa JA, Touchon M, Rocha EPC. 2021. Bacteria have numerous distinctive groups of phage–plasmids with conserved phage and variable plasmid gene repertoires. Nucleic Acids Res 49:2655–2673.

2. Brown CL, Mullet J, Hindi F, Stoll JE, Gupta S, Choi M, Keenum I, Vikesland P, Pruden A, Zhang L. 2022. mobileOG-db: a Manually Curated Database of Protein Families Mediating the Life Cycle of Bacterial Mobile Genetic Elements. Appl Environ Microbiol 88.

3. Pfeifer, E., Rocha, E.P.C. Phage-plasmids promote recombination and emergence of phages and plasmids. Nat Commun 15, 1545 (2024). https://doi.org/10.1038/s41467-024-45757-3

4. Schmartz GP, Hartung A, Hirsch P, Kern F, Fehlmann T, Müller R, Keller A. 2022. PLSDB: advancing a comprehensive database of bacterial plasmids. Nucleic Acids Res 50:D273–D278.

5. Camargo AP, Nayfach S, Chen IMA, Palaniappan K, Ratner A, Chu K, Ritter SJ, Reddy TBK, Mukherjee S, Schulz F, Call L, Neches RY, Woyke T, Ivanova NN, Eloe-Fadrosh EA, Kyrpides NC, Roux S. 2023. IMG/VR v4: an expanded database of uncultivated virus genomes within a framework of extensive functional, taxonomic, and ecological metadata. Nucleic Acids Res 51:D733–D743.

6. Steinegger M, Söding J. 2017. MMseqs2 enables sensitive protein sequence searching for the analysis of massive data sets. Nat Biotechnol 2017 3511 35:1026–1028.

7. Nayfach S, Páez-Espino D, Call L, Low SJ, Sberro H, Ivanova NN, Proal AD, Fischbach MA, Bhatt AS, Hugenholtz P, Kyrpides NC. 2021. Metagenomic compendium of 189,680 DNA viruses from the human gut microbiome. Nat Microbiol 2021 67 6:960–970.

8. Li W, Godzik A. 2006. Cd-hit: a fast program for clustering and comparing large sets of protein or nucleotide sequences. Bioinformatics 22:1658–1659.

9. Jain C, Rodriguez-R LM, Phillippy AM, Konstantinidis KT, Aluru S. 2018. High throughput ANI analysis of 90K prokaryotic genomes reveals clear species boundaries. Nat Commun 2018 91 9:1–8.

10. Arango-Argoty G, Garner E, Pruden A, Heath LS, Vikesland P, Zhang L. 2018. DeepARG: A deep learning approach for predicting antibiotic resistance genes from metagenomic data. Microbiome 6:1–15.

11. Botelho J. 2023. Defense systems are pervasive across chromosomally integrated mobile genetic elements and are inversely correlated to virulence and antimicrobial resistance. Nucleic Acids Res 51:4385–4397.

12. Ruiz-Perez CA, Conrad RE, Konstantinidis KT. 2021. MicrobeAnnotator: a user-friendly, comprehensive functional annotation pipeline for microbial genomes. BMC Bioinformatics 22:1–

13. Bate N, Cundliffe E. 1999. The mycinose-biosynthetic genes of Streptomyces fradiae, producer of tylosin. J Ind Microbiol Biotechnol 23:118–122.

14. Meta Cyc: Metabolic Pathways From all Domains of Life. https://metacyc.org/. Retrieved 3 December 2023.

15. Kanehisa M, Sato Y, Kawashima M, Furumichi M, Tanabe M. 2016. KEGG as a reference resource for gene and protein annotation. Nucleic Acids Res 44:D457.

16. Thuy TTT, Liou K, Oh TJ, Kim DH, Nam DH, Yoo JC, Sohng JK. 2007. Biosynthesis of dTDP-6-deoxy-β-d-allose, biochemical characterization of dTDP-4-keto-6-deoxyglucose reductase (GerKI) from Streptomyces sp. KCTC 0041BP. Glycobiology 17:119–126.

17. donovan-h-parks/CompareM: A toolbox for comparative genomics. https://github.com/donovan-h-parks/CompareM. Retrieved 22 October 2023.

18. SankeyMATIC: Make Beautiful Flow Diagrams. https://sankeymatic.com/. Retrieved 10 December 2023.

19. Camargo AP, Roux S, Schulz F, Babinski M, Xu Y, Hu B, Chain PSG, Nayfach S, Kyrpides NC. 2023. Identification of mobile genetic elements with geNomad. Nat Biotechnol 2023 1–10.

20. Carattoli A, Zankari E, Garciá-Fernández A, Larsen MV, Lund O, Villa L, Aarestrup FM, Hasman H. 2014. In silico detection and typing of plasmids using PlasmidFinder and plasmid multilocus sequence typing. Antimicrob Agents Chemother 58:3895–3903.

21. Yu MK, Fogarty EC, Eren AM. 2024. Diverse plasmid systems and their ecology across human gut metagenomes revealed by PlasX and MobMess. Nat Microbiol 2024 93 9:830–847.

22. Shannon P, Markiel A, Ozier O, Baliga NS, Wang JT, Ramage D, Amin N, Schwikowski B, Ideker T. 2003. Cytoscape: A Software Environment for Integrated Models of Biomolecular Interaction Networks. Genome Res 13:2498.

23. Seemann T. 2014. Prokka: rapid prokaryotic genome annotation. Bioinformatics 30:2068–2069.

24. Grant JR, Enns E, Marinier E, Mandal A, Herman EK, Chen CY, Graham M, Van Domselaar G, Stothard P. 2023. Proksee: in-depth characterization and visualization of bacterial genomes. Nucleic Acids Res 51:W484–W492.

25. Starikova E V., Tikhonova PO, Prianichnikov NA, Rands CM, Zdobnov EM, Ilina EN, Govorun VM. 2020. Phigaro: high-throughput prophage sequence annotation. Bioinformatics 36:3882–3884.

26. Couvin D, Bernheim A, Toffano-Nioche C, Touchon M, Michalik J, Néron B, Rocha EPC, Vergnaud G, Gautheret D, Pourcel C. 2018. CRISPRCasFinder, an update of CRISRFinder, includes a portable version, enhanced performance and integrates search for Cas proteins. Nucleic Acids Res 46:W246–W251.

27. Payne LJ, Meaden S, Mestre MR, Palmer C, Toro N, Fineran PC, Jackson SA. 2022. PADLOC: a web server for the identification of antiviral defence systems in microbial genomes. Nucleic Acids Res 50:W541–W550.

28. Pal C, Bengtsson-Palme J, Rensing C, Kristiansson E, Larsson DGJ. 2014. BacMet: antibacterial biocide and metal resistance genes database. Nucleic Acids Res 42:D737.

29. Liu B, Zheng D, Zhou S, Chen L, Yang J. 2022. VFDB 2022: a general classification scheme for bacteria

30. Ilchenko K, Bonnin RA, Rocha EPC, Pfeifer E. 2026. Efficient detection and typing of phage-plasmids. mBio 17:e03000-25.

31. Pfeifer E, Bonnin RA, Rocha EPC. 2022. Phage-Plasmids Spread Antibiotic Resistance Genes through Infection and Lysogenic Conversion. MBio 13.
